# Activation of mGlu3 Receptors Stimulates the Production of GDNF in Striatal Neurons

**DOI:** 10.1371/journal.pone.0006591

**Published:** 2009-08-12

**Authors:** Giuseppe Battaglia, Gemma Molinaro, Barbara Riozzi, Marianna Storto, Carla L. Busceti, Paola Spinsanti, Domenico Bucci, Valentina Di Liberto, Giuseppina Mudò, Corrado Corti, Mauro Corsi, Ferdinando Nicoletti, Natale Belluardo, Valeria Bruno

**Affiliations:** 1 Istituto Neurologico Mediterraneo Neuromed, Pozzilli, Italy; 2 Department of Human Physiology and Pharmacology, University “La Sapienza”, Rome, Italy; 3 DIMES, Human Physiology Section, University of Palermo, Palermo, Italy; 4 Neuroscience Centre of Excellence in Drug Discovery, GlaxoSmithKline Medicines Research Centre, Verona, Italy; Tokyo Medical and Dental University, Japan

## Abstract

Metabotropic glutamate (mGlu) receptors have been considered potential targets for the therapy of experimental parkinsonism. One hypothetical advantage associated with the use of mGlu receptor ligands is the lack of the adverse effects typically induced by ionotropic glutamate receptor antagonists, such as sedation, ataxia, and severe learning impairment. Low doses of the mGlu2/3 metabotropic glutamate receptor agonist, LY379268 (0.25–3 mg/kg, i.p.) increased glial cell line-derived neurotrophic factor (GDNF) mRNA and protein levels in the mouse brain, as assessed by *in situ* hybridization, real-time PCR, immunoblotting, and immunohistochemistry. This increase was prominent in the striatum, but was also observed in the cerebral cortex. GDNF mRNA levels peaked at 3 h and declined afterwards, whereas GDNF protein levels progressively increased from 24 to 72 h following LY379268 injection. The action of LY379268 was abrogated by the mGlu2/3 receptor antagonist, LY341495 (1 mg/kg, i.p.), and was lost in mGlu3 receptor knockout mice, but not in mGlu2 receptor knockout mice. In pure cultures of striatal neurons, the increase in GDNF induced by LY379268 required the activation of the mitogen-activated protein kinase and phosphatidylinositol-3-kinase pathways, as shown by the use of specific inhibitors of the two pathways. Both *in vivo* and *in vitro* studies led to the conclusion that neurons were the only source of GDNF in response to mGlu3 receptor activation. Remarkably, acute or repeated injections of LY379268 at doses that enhanced striatal GDNF levels (0.25 or 3 mg/kg, i.p.) were highly protective against nigro-striatal damage induced by 1-methyl-4-phenyl-1,2,3,6-tetrahydropyridine in mice, as assessed by stereological counting of tyrosine hydroxylase-positive neurons in the pars compacta of the substantia nigra. We speculate that selective mGlu3 receptor agonists or enhancers are potential candidates as neuroprotective agents in Parkinson's disease, and their use might circumvent the limitations associated with the administration of exogenous GDNF.

## Introduction

Metabotropic glutamate (mGlu) receptors have been considered potential targets for neuroprotective drugs since the early times of their characterization. One hypothetical advantage associated with the use of mGlu receptor ligands is the lack of the adverse effects typically induced by N-metyl-D-aspartate (NMDA) or α-amino-3-hydroxy-5-methyl-4-isoxazolepropionate (AMPA) receptor antagonists, such as sedation, ataxia, and severe learning impairment [1], [2]. mGlu receptors form a family of eight subtypes (mGlu1 to −8), subdivided into three groups on the basis of their amino acid sequence, pharmacological profile and transduction pathways. Group-II mGlu receptors (including subtypes mGlu2 and mGlu3) are best candidates as “neuroprotective receptors” because their activation inhibits glutamate release [3], [4], [5], [6,], inhibits voltage-gated calcium channels [7], positively modulates potassium channels [8], and stimulates the production of neurotrophic factors in astrocytes and microglia [9], [10], [11], [12], [13]. The use of mixed cell cultures containing both neurons and astrocytes has shown that activation of glial mGlu3 receptors enhances the formation of transforming-growth factor-β (TGF-β), which in turn protects neighbor neurons against excitotoxic death [9], [10], [12], [14,]. This raises the intriguing possibility that pharmacological activation of particular mGlu receptor subtypes may slow the progression of neurodegenerative disorders through a non conventional mechanism based on the production of endogenous neurotrophic factor. A recent review highlights the potential role of mGlu receptors in the experimental treatment of Parkinson's disease [15], in which only symptomatic drugs are currently used. A particular advantage of subtype-selective mGlu receptor ligands (such as mGlu2/3 receptor agonists, mGlu4 receptor enhancers, or mGlu5 receptor antagonists) is that these drugs not only relieve motor symptoms, but are also protective against nigro-striatal damage at least in experimental animal models of parkinsonism [13], [16], [17], [18], [19], [20], [21]. Along this line, we decided to study whether activation of group-II mGlu receptors influences the endogenous production of glial cell line-derived neurotrophic factor (GDNF), which is a potent factor for survival and axonal growth of mesencephalic dopaminergic neurons and has been shown to improve motor symptoms and attenuate nigro-striatal damage in experimental animal models of parkinsonism [22], [23], [24], [25], [26]. Several clinical trial have evaluated the efficacy of intraputaminal infusion of GDNF in Parkinsonian patients with contrasting results (see Discussion and references therein). Interestingly, the protective activity of GDNF in the 1-methyl-4-phenyl-1,2,3,6-tetrahydropyridine (MPTP) model of parkinsonism requires the presence of TGF-β [27], suggesting that strategies aimed at enhancing the endogenous production of both GDNF and TGF-β may be particularly successful in slowing the progression of Parkinson's disease.

We now report that selective pharmacological activation of mGlu3 receptors enhances the production of GDNF in mouse striatum, and that the potent mGlu2/3 receptor agonist, LY379268, is highly protective in the MPTP model of parkinsonism at doses that up-regulate GDNF.

## Results

### 1. Pharmacological activation of mGlu3 receptors enhances GDNF formation in the striatum

**Figure 1:**
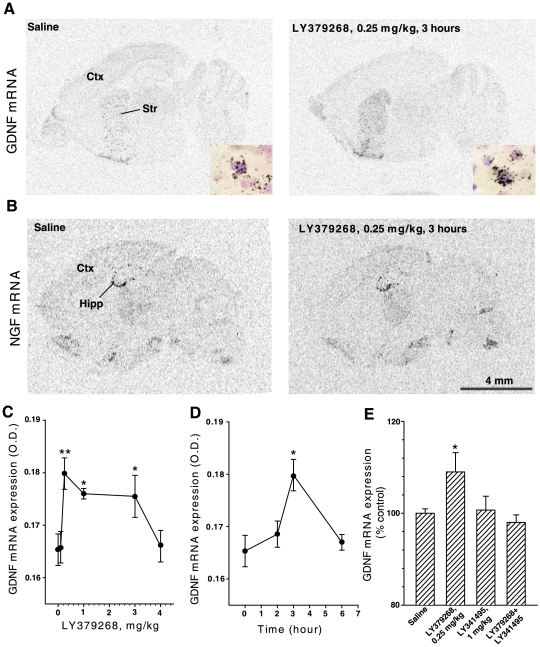
*In situ* hybridization of sagittal sections at basal ganglia level showing the expression of mRNA encoding GDNF (A) or NGF (B). Autoradiogram showing GDNF expression in the striatum of saline-treated mice or LY379268 (0.25 mg/kg, i.p.)-treated mice (A). The inserts show representative GDNF mRNA labeled cells (black grains) with increased levels of labeling in LY379268-treated mice. Autoradiogram showing NGF expression of saline-treated mice or LY379268 (0.25 mg/kg, i.p.)-treated mice (B). Dose-response curve of GDNF mRNA levels in the striatum of mice treated with saline or LY379268 (0.1, 0.25, 1, 3 or 4 mg/kg, i.p) (C) and time-course of GDNF mRNA levels in the striatum of mice after a single injection of LY379268 (0.25 mg/kg, i.p.) (D); values are means±S.E.M (n = 4–5, animals per group; three independent experiments). Striatal GDNF mRNA levels in mice treated with saline, LY379268 (0.25 mg/kg, i.p), LY341495 (1 mg/kg, i.p) or LY379268+LY341495 (E); value are means±S.E.M (n = 4, animals per group; three independent experiments). *p<0.05; **p<0.01 (One-way ANOVA+Fisher's PLSD) vs. control mice. Scale bar: A–B = 4 mm. Str, striatum; Ctx, cortex; Hipp, hippocampus.

**Figure 2:**
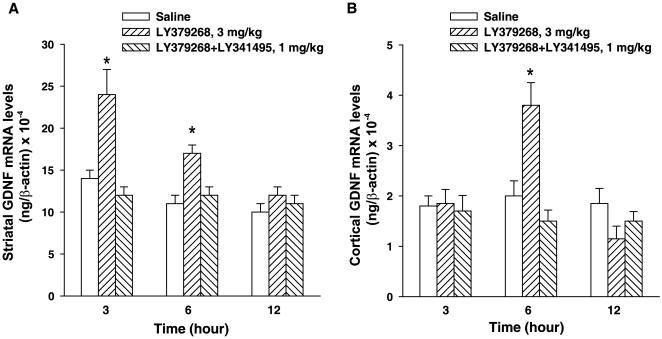
Quantitative real-time PCR analysis of GDNF mRNA in mouse striatum (A) and cortex (B) at 3, 6 or 12 h after systemic treatment with saline, LY379268 (3 mg/kg, i.p.), or LY379268 (3 mg/kg, i.p.)+LY341495 (1 mg/kg, i.p.). Values were normalized with respect to the amount of β-actin mRNA. Values are mean+S.E.M. of four determinations (each from triplicates). *p<0.05 (One-way ANOVA+Fisher's PLSD) vs. saline-treated mice.

**Figure 3:**
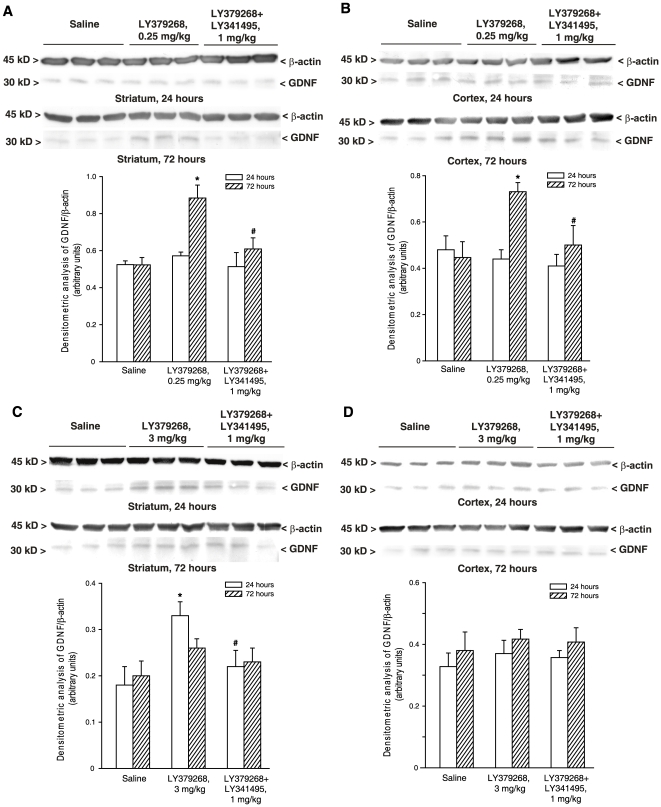
Western blot analysis of GDNF expression in the striatum (A,C) or cerebral cortex (B,D) of mice after treatment with saline, LY379268, 0.25 (A,B) or 3 (C,D) mg/kg, i.p., or LY379268+LY341495, 1 mg/kg, i.p. Animals were killed 24 or 72 h after treatments. Densitometric data of GDNF are shown and are the mean+S.E.M. of 3 animals performed two times.*p<0.05 (One-way ANOVA+Fisher's PLSD) vs. saline-treated mice.

**Figure 4:**
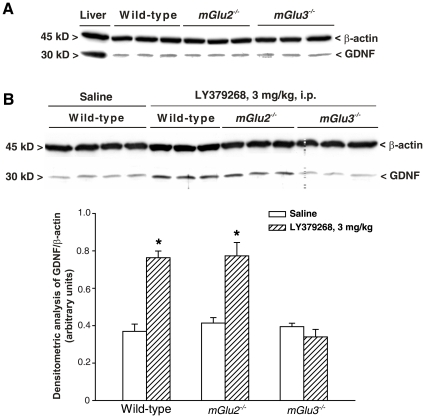
Western blot analysis of striatal GDNF expression in wild-type, *mGlu2^−/−^* or *mGlu3^−/−^* mice in basal conditions (A) and after treatment with LY379268, 3 mg/kg, i.p. (B). Animals were killed 24 h later. Densitometric data of GDNF are shown and are the mean+S.E.M. of 3 animals performed two times.*p<0.05 (One-way ANOVA+Fisher's PLSD) vs. saline-treated mice.

Mice were systemically injected with LY379268, a drug that selectively activates mGlu2/3 receptors with nanomolar potency and is systemically active [28]. *In situ* hybridization analysis showed that LY379268 treatment increased GDNF mRNA levels in the striatum (Fig. 1A), but had no effect on NGF mRNA (Fig. 1B). LY379268 treatment increased the amount of GDNF mRNA, evaluated as number of grains per cell (saline = 25.96±1.1 vs LY379268 = 32.35±0.71, p<0.002) without affecting the number of GDNF-mRNA positive cells (not shown). Dose-dependent experiments showed an inverse-U shaped dose-response curve, with maximal responses at 0.25 mg/kg of LY37968, a plateau between 0.25 and 3 mg/kg, and loss of response at 4 mg/kg, i.p. (Fig. 1C). This is remarkable because LY379268 is usually administered to mice at systemic doses>0.3–0.5 mg/kg [18], [29], [30], [31], [32], [33]. The increase in striatal GDNF mRNA levels induced by a single injection of LY379268 peaked after 3 h (Fig. 1D) and was prevented by the preferential mGlu2/3 receptor antagonist, LY341495 (1 mg/kg, i.p.), which had no effect on its own (Fig. 1E). Quantitative analysis by real-time PCR confirmed the increase in GDNF mRNA induced by LY379268 at 3 h and showed a residual effect at 6 h that was not detected by *in situ* hybridisation analysis (Fig. 2A). In addition, real-time PCR analysis revealed an effect of LY379268 on GDNF mRNA levels in the cerebral cortex, which, however, was only detected at 6 h (note that GDNF levels are 10-fold lower in the cerebral cortex than in the striatum) (Fig. 2B). We extended the study to GDNF protein levels, which we measured by immunoblotting in striatal and cortical extracts from mice treated with LY379268. Treatment with 0.25 mg/kg LY379268 increased GDNF protein levels in both the striatum and cerebral cortex after 72 h (Fig. 3A,B). When mice were treated with 3 mg/kg LY379268, GDNF levels increased in the striatum after 24 h and returned back to normal at 72 h (Fig. 3C). All these effects were prevented by the antagonist, LY341495 (Fig. 3A–C). Interestingly, the high dose of LY379268 had no effect on GDNF levels in the cerebral cortex (Fig. 3D). To unravel the identity of the mGlu receptor subtype that mediates the increase in GDNF levels, we administered LY379268 (3 mg/kg, i.p.) to mice lacking either mGlu2 or mGlu3 receptors, and examined GNDF levels in the striatum 24 h later. Basal GDNF levels did not differ among wild-type, *mGlu2^−/−^* and *mGlu3^−/−^* mice (Fig. 4A). In contrast, treatment with LY379268 was able to enhance GDNF levels in wild-type and *mGlu2^−/−^* mice, but not in *mGlu3^−/−^* mice (Fig. 4B).

### 2. The increase in GDNF mediated by mGlu3 receptors selectively occurs in neurons

**Figure 5:**
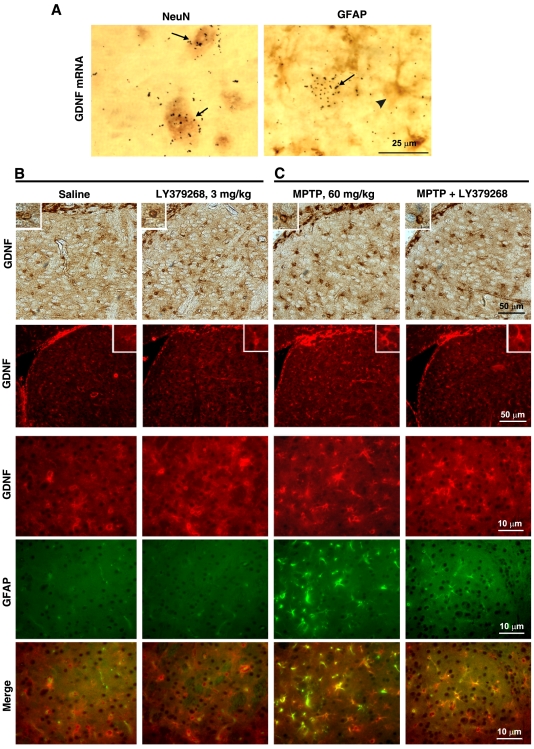
Double immunolabeling for GDNF and NeuN or GFAP in striatal cells showing the labelling of GDNF within neuronal cells (A). Arrows in the left panel, NeuN-positive cells containing GDNF mRNA black grains; arrow in the right panel, GDNF mRNA black grains, arrow head in the right panel, GFAP-positive cell. Immunohistochemical analysis of GDNF in the striatum of mice treated with a single injection of LY379268 (3 mg/kg, i.p.) and killed 24 h later (B). In both control mice and mice treated with LY379268, GDNF immunoreactivity is exclusively localized in neurons (note the absence of co-localization between GDNF and GFAP), and the extent of immunostaining increases after drug treatment. GDNF immunostaining in the striatum of mice treated 7 days before with MPTP, 20 mg/kg, i.p., x 3, two h apart (C). This treatment led to reactive gliosis in the striatum, as a result of the degeneration of nigro-striatal dopaminergic neurons. Under these conditions, GDNF immunostaining is localized both in neurons and reactive astrocytes. A single injection of LY379268 (3 mg/kg, i.p.) 7 days following MPTP injection did not enhance GDNF immunoreactivity in reactive astrocytes, but still enhanced immunoreactivity in neurons. Interestingly, the number of GDNF-positive reactive astrocytes is lower 24 h following LY379268 injection. Scale bar = 50 and 10 µm.

A combination of *in vivo* and *in vitro* experiments clearly showed that the source of the GDNF responsive to mGlu3 receptor activation was exclusively neuronal. Double labelling analysis by combined *in situ* hybridization and immunohistochemistry (GDNF mRNA+NeuN or GFAP) showed that GDNF is expressed in neurons (Fig. 5A) and treatment with LY379268 (0.25 mg/kg, i.p., 3 h) selectively increased GDNF mRNA levels in neurons (not shown). GDNF immunostaining was also performed in the striatum of mice treated 7 days before with high doses of the parkinsonian toxin, MPTP (20 mg/kg, i.p., x 3, two h apart). This treatment led to reactive gliosis in the striatum, as a result of the degeneration of nigro-striatal dopaminergic neurons (see GFAP immunostaining in Fig. 5C). Under these conditions, GDNF immunostaining was localized both in neurons and reactive astrocytes. A single injection of LY379268 (3 mg/kg, i.p.) 7 days following MPTP injection did not enhance GDNF immunoreactivity in reactive astrocytes, but still enhanced immunoreactivity in neurons. Interestingly, the number of GDNF^+^ reactive astrocytes was even less 24 h following LY379268 injection (Fig. 5B).

### 3. Activation of mGlu3 receptors enhances GDNF levels in cultured striatal neurons (but not in astrocytes) via the activation of the MAP kinase and the phosphatidylinositol-3-kinase pathways

**Figure 6:**
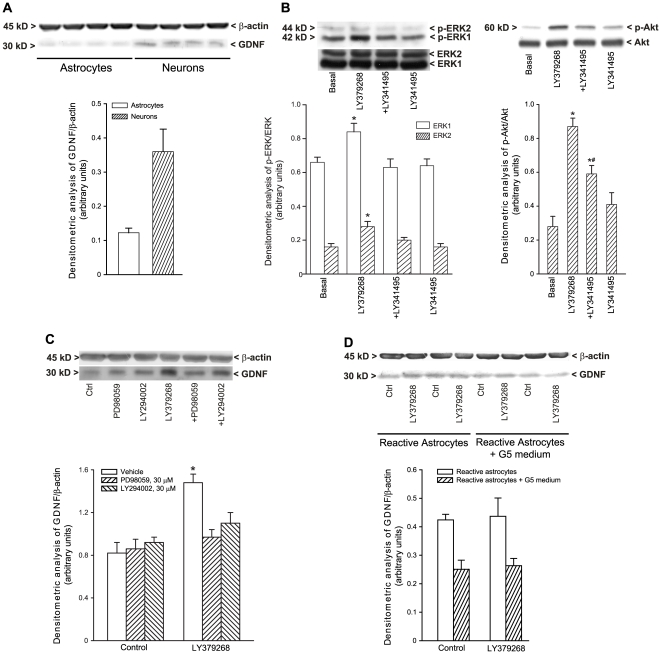
Basal GDNF levels in cultured mouse striatal neurons and in cultured astrocytes (A). Expression of phosphoERK1/2 and phospho-Akt in cultured striatal neurons treated with LY379268 (1 µM), LY341495 (1 µM) and LY379268+LY341495 for 15 min (B). Densitometric values are means+S.E.M. of 3–4 determinations. *p<0.05 (One-Way ANOVA+Fisher's PLSD) vs. basal values, #p<0.05 vs. LY379268 values. Treatment of cultured neurons with 1 µM LY379268 enhanced GDNF levels 24 h later (C), and it was abrogated by the co-application of the MEK inhibitor, PD98059, or the PI-3-K inhibitor, LY294002 (C). Application of LY379268 to astrocytes made “reactive” by several passages in culture and by the G5 supplement in the medium did not affect GDNF levels (D).

In *in vitro* studies, basal GDNF levels were about 3 fold higher in cultured mouse striatal neurons than in cultured astrocytes (Fig. 6A). Treatment of cultured neurons with 1 µM LY379268 enhanced GDNF levels 24 h later. This effect was abrogated by a co-application of the MEK inhibitor, PD98059 (30 µM), or the phosphatidyilinositol-3-kinase (PI-3-K) inhibitor, LY294002 (30 µM) (Fig. 6C). As expected, LY379268 activated both the MAP kinase and the PI-3-K pathways, as shown by an increased levels of phosphorylated ERK1/2 and phosphorylated Akt, respectively. The action of LY379268 was abrogated by the antagonist, LY341495 (1 µM) (Fig. 6B). Application of LY379268 to quiescent astrocytes (not shown) or astrocytes made “reactive” by several passages in culture and by the G5 supplement in the medium did not affect GDNF levels (Fig. 6D).

### 4. Doses of LY379268 that are effective in enhancing GDNF levels protect nigro-striatal neurons against MPTP toxicity in mice

**Figure 7:**
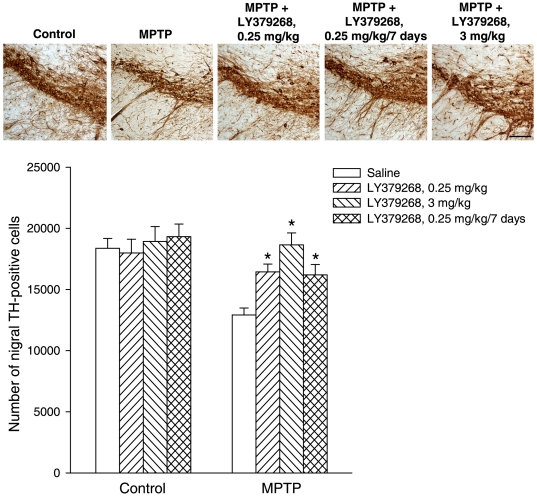
Immunohistochemical analysis of TH in the pars compacta of substantia nigra of mice injected with a single i.p. dose of 30 mg/kg of MPTP, alone or combined with LY379268 (0.25 or 3 mg/kg in a single i.p. injection, 30 min prior to MPTP injection or 0.25 mg/kg/7 days once a day, i.p.). Scale bar = 100 µm. Stereological TH-positive cell counts are also shown. Values (means+S.E.M.) were calculated from 7–8 mice per group (10 sections - 10 µm thick, cut every 100 µm, per animal were used for the calculation of the density of TH-positive neurons in the pars compacta of the substantia nigra). *p<0.05 (One-way ANOVA+Fisher's PLSD) vs. mice treated with MPTP alone.

**Figure 8:**
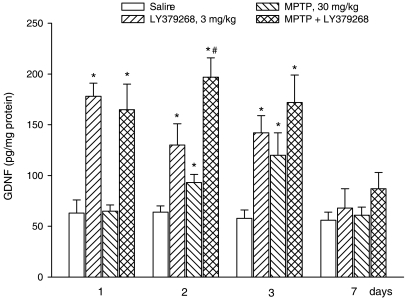
ELISA analysis of GDNF expression in the striatum of mice after treatment with saline, LY379268 (3 mg/kg, i.p.), MPTP (30 mg/kg, i.p.), or MPTP+LY379268. Animals were killed 1,2,3 or 7 days after treatments. Data of GDNF are the mean+S.E.M. of 8 animals. *p*<0.05 (One-way ANOVA+Fisher's PLSD) vs. the corresponding groups of mice treated with saline (*) or with MPTP or LY379268 alone (#).

**Figure 9:**
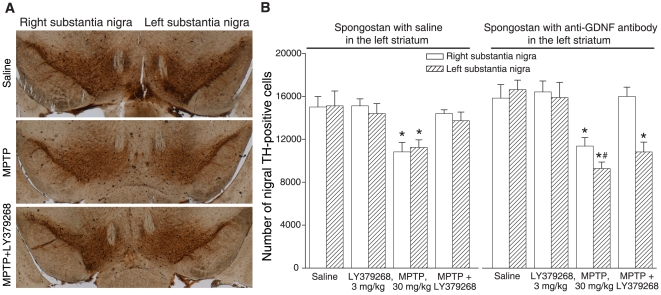
LY379268 fails to protects against MPTP toxicity in mice unilaterally implanted with anti-GDNF antibodies. Mice were implanted with a gelfoam (Spongostan) pre-soaked with saline alone (A,B) or a saline solution containing 5 µg of neutralizing anti-GDNF antibodies (A,B) in the left caudate nucleus. Stereological counts of TH-positive neurons in the substantia nigra pars compact in the implantation side (left) or contralateral side (right) in response to i.p. injection of saline, LY379268 (3 mg/kg), MPTP alone (30 mg/kg) or MPTP+LY379268 (injected 30 min prior to MPTP injection). Drugs were administered 24 h after the gelfoam implantation. Mice were killed 7 days after MPTP injection. Values (means+S.E.M.) were calculated from 6 mice per group. *p*<0.05 (One-way ANOVA+Fisher's PLSD) vs. the corresponding values in mice treated with saline (*) or vs. the MPTP values of the right side (#).

We decided to examine whether doses of 0.25 or 3 mg/kg of LY379268, which enhanced GDNF levels in the striatum, were protective against nigro-striatal damage in mice treated with MPTP. We used a dose of MPTP (30 mg/kg, single i.p. injection) that causes about a 40–50% degeneration of nigro-striatal dopaminergic neurons, and is known to be insensitive to higher doses of LY379268 (5 or 10 mg/kg, i.p.) [18]. The number of nigral neurons was assessed by stereological counting 7 days following MPTP injection. Mice receiving either 0.25 or 3 mg/kg LY379268 30 min prior to MPTP injection or mice receiving 0.25 mg/kg LY379268 once a day for 7 days starting from the day of MPTP injection were significantly protected against nigro-striatal damage. In mice treated with MPTP alone there was a loss of about 35% of neurons in the pars compacta of the substantia nigra. The number of surviving neurons substantially increase in mice receiving either a single or a repeated injection with 0.25 mg/kg LY379268, and returned back to normal in mice receiving a single injection with 3 mg/kg LY379268 (Fig. 7). Searching for a correlation between GDNF and neuroprotection, we measured GDNF levels in response to the most effective dose of LY379268 (3 mg/kg, single injection) given alone or in combination with MPTP. In control mice, striatal GDNF levels were approximately 55–65 pg/mg protein, and were relatively stable at 1, 2, 3, and 7 days after the injection of saline. A single injection of LY379268 alone enhanced GDNF levels by more than 2 fold after 1 day. The increase was still visible at 2 days and became negligible at 3 and 7 days. MPTP alone did not change GDNF levels at 1 day, but enhanced GDNF levels at 2 and 3 days, perhaps as a result of reactive gliosis (see above). Interestingly, the combination of LY379268 further enhanced the increase in GDNF levels induced by MPTP at 2 and 3 days (Fig. 8). To examine the causal relationship between the increase in GDNF levels and neuroprotection, we used mice unilaterally implanted with gelfoam (Spongostan) pre-adsorbed with saline alone (controls) or with saline containing 5 µg of neutralizing anti-GDNF antibodies into the left caudate nucleus. In control mice, systemic injection of LY379268 (3 mg/kg, i.p.) protected nigro-striatal neurons against MPTP toxicity, as expected (Fig. 9A, B). In mice implanted with Spongostan containing anti-GDNF antibodies, MPTP caused a greater loss of TH-positive nigral neurons in the implantation side (left side). Interestingly, treatment with LY379268 was still protective against MPTP toxicity in the side contralateral to implantation (right side), but lost its protective activity in the implantation side (Fig. 9A, B).

## Discussion

The evidence that the mGlu2/3 receptor agonist, LY404039, relieves psychotic symptoms in schizophrenic patients [34] has renewed the interest on group-II mGlu receptors. However, it is the mGlu2 receptor that mediates the antipsychotic activity of mGlu2/3 receptor agonists [33], [35], [36], while the mGlu3 receptor is still in search of a function that can be relevant for human studies. Using mixed cultures of mouse cortical cells, we have found that the protective activity of LY379268 against excitotoxic neuronal death is entirely mediated by the mGlu3 receptor [14]. Activation of mGlu3 receptors present in astrocytes enhances the production of TGF-β, which in turn protects neighbour neurons against excitotoxicity [10], [12]. Activation of mGlu3 receptors can also enhance the production of nerve growth factor (NGF) and S-100β in cultured astrocytes [11], and group-II mGlu receptor agonists stimulate the secretion of brain-derived neurotrophic factor from cultured microglia [13], [16]. It seems therefore that the mGlu3 receptor is endowed with the unusual activity of regulating the production of neurotrophic factors in glial cells. We have now extended this function to neurons, where activation of mGlu3 receptors stimulated GDNF production. This particular activity was prominent in striatal neurons, which are a major source of GDNF in the forebrain [37], [38]. Interestingly, the orthosteric mGlu2/3 receptor agonist, LY379268, displayed an unusually high potency in stimulating GDNF production in the striatum of living mice, with doses as low as 0.25 mg/kg, displaying maximal activity, and doses≥4 mg/kg being inactive. There are a number of potential explanations for the lack of activity of high doses of LY39268, which include the recruitment of additional mGlu receptor subtypes, such as mGlu2 and mGlu8 receptors [39], the recruitment of additional intracellular pathways that negatively regulate the transcriptional machinery of the GDNF gene, or the development of tachyphylaxis. Using pure cultures of striatal neurons we could establish that activation of mGlu3 receptors enhanced GDNF production by stimulating the MAPK and the PI-3-K pathways. This was expected because the MAPK and PI-3-K pathways mediate the effect of mGlu3 receptor activation on TGF-β production in cultured astrocytes [12]. Perhaps it is not a mere coincidence that the same receptor positively regulates the formation of GDNF and TGF-β. GDNF and other GDNF-family ligands, such as neurturin, artemin, and persephin, belong to the TGF-β superfamily and share with TGF-β the protein conformation and the ability to function as homodimers [40]. Interestingly, GDNF and TGF-β act synergistically as neuroprotectants although the respective receptors do not share the same transduction machinery. For example, endogenous TGF-β is required for the ability of GDNF to rescue target-derived sympathetic spinal cord neurons [41], and antibodies neutralizing the three isoforms of TGF-β abolish the protective activity of GDNF against MPTP-induced nigro-striatal lesions in mice [27]. Although the molecular nature of the cross-talk between GDNF and TGF-β is unknown, drugs that enhance the production of both factors are promising candidates as neuroprotective agents. GDNF was shown to behave as a potent neurotrophic factor for midbrain dopaminergic neurons since the time of its discovery [22]. Interestingly, TGF-β also behaves as a survival factor for midbrain dopaminergic neurons [42]. Moving from the consistent neuroprotective and restoring activity in experimental models of parkinsonism (see references in Introduction), GDNF has been evaluated in several clinical trials for its effect on Parkinson's disease. Intracerebroventricular injection of GDNF was inactive [43], whereas direct intraputaminal infusion of GDNF produced beneficial effects in two phase-I clinical trials [44], [45], but was not successful in another double-blinded placebo controlled study [46]. The use of brain-permeant drugs that mimic the action of GDNF or enhance the production of endogenous GDNF is an attractive strategy that may overcome the limitations associated with an invasive delivery of GDNF into the brain. For example, the orally active compound, PYM50028, which elevates striatal levels of GDNF, is protective against MPTP toxicity in mice [47], and induction of GDNF is associated with the neurorescue action of rasagilin, a drug of current use in the treatment of Parkinson's disease [48], [49]. Brain-permeant mGlu2/3 receptor agonists are particularly promising for the experimental treatment of Parkinson's disease for the ability to enhance striatal levels of GDNF (present data) and TGF-β [12], [14]. The ability of GDNF-enhancing doses of LY379268 to protect nigro-striatal neurons against MPTP toxicity supports the use of mGlu2/3 receptor agonists as neuroprotectants in Parkinson's disease. An important question is whether the amount of GDNF produced in response to LY379268 was sufficient to afford neuroprotection against MPTP toxicity. In control mice, striatal GDNF levels were about 60 pg/mg prot., a value similar to that reported in [47]. A single injection of LY379268 increased striatal GDNF levels by about 80–100 pg/mg protein after 48–72 hours. These are steady-state levels that reflect the equilibrium between production and clearance/degradation of GDNF, and do not discriminate between intracellular and extracellular GDNF. A direct demonstration that these steady-state levels of GDNF are neuroprotective requires accurate titration experiments with viral vectors, and is technically difficult. However, it is remarkable that neuroprotective doses of compound PYM50028, which is in clinical development for the treatment of Parkinson's disease, induce increases in striatal GDNF levels in MPTP-treated mice similar to those induced by LY379268 in our study [47]. We were unable to use mutant mice lacking GDNF to examine the direct link between GDNF and the protective activity of LY379268 against MPTP toxicity. Thus, we adopted an alternative strategy based on the implantation of neutralizing anti-GDNF antibodies in the caudate nucleus. We adopted the same experimental protocol developed in [27], based on the unilateral implantation of a gelfoam adsorbed with the antibody. The loss of nigral neurons in response to MPTP was slightly greater in the implantation side, suggesting that endogenous production of GDNF is protective against MPTP toxicity. Interestingly, LY379268 lost its protective activity in the implantation side, lending credit to the hypothesis that mGlu3 receptor activation protects *via* an increase in striatal GDNF levels. The presence of the anti-GDNF antibodies was the critical determinant in these experiments because LY379268 was still protective when a gelfoam lacking the antibody was implanted in the striatum.

In conclusion, we have shown that activation of mGlu3 receptors enhances GDNF levels in neurons, and that doses of a mGlu2/3 receptor agonist that enhance GDNF levels in the striatum are protective against MPTP-induced nigro-striatal damage. This finding is particularly interesting because pharmacological activation of mGlu2/3 receptors can also improve motor deficits in experimental models of parkinsonism [50], [51]. The good profile of safety and tolerability of mGlu2/3 receptor agonists in clinical studies [34] may encourage the use of mGlu2/3 receptor agonists in the experimental treatment of Parkinson's disease.

## Materials and Methods

(-)-2-Oxa-4-aminobicyclo[3.1.0]hexane-4,6-dicarboxylic acid (LY379268) was kindly provided by Eli Lilly Research Laboratories (Indianapolis, IN). (2S)-2-Amino-2-[(1S,2S)-2-carboxycycloprop-1-yl]-3-(xanth-9-yl) propanoic acid (LY341495) was purchased from Tocris Cookson Ltd. (Bristol, UK). All other chemicals were purchased from Sigma (Milano, Italy).

### mGlu2 and mGlu3 receptor knockout mice

mGlu2 receptor knockout mice (*mGlu2^−/−^*) were obtained from University of Kyoto, Japan [52]. mGlu3 receptor knockout mice (*mGlu3^−/−^*) were generated by GlaxoSmithKline, Verona, Italy [14]. Mice were backcrossed up to the 17th generation on C57BL/6J genetic background and bred in a specific pathogen-free (SPF) breeding colony.

### Preparation of mouse striatal cultures

Glial cell cultures were prepared from striatum of postnatal C57 Black mice (1–3 days after birth), as previously described [53]. Dissociated striatal cells were grown in 100 mm dishes (Falcon Primaria, Lincoln Park, NJ) using a plating medium of MEM-Eagle's salts supplemented with 10% of heat inactivated horse serum, 10% fetal bovine serum, 2 mM glutamine, 25 mM sodium bicarbonate and 21 mM glucose. Cultures were kept at 37°C in a humidified CO_2_ atmosphere. After confluence had been reached, the cells in each dish were dissociated by trypsin treatment and plated in new dishes. In one set of experiments cell differentiation was initiated by decreasing the foetal bovine serum concentration to 3% and the culture medium was also supplemented for 10 days with the defined culture additive G5 medium diluted 1/100, as suggested by the manufacturer (composition: insulin 500 mg/ml, human transferrin 5000 mg/ml, selenite 0.52 mg/ml, biotin 1 mg/ml, hydrocortisone 0.36 mg/ml, FGF2 0.52 mg/ml and EGF 1 mg/ml) in order to trigger astrocyte activation. After confluence had been reached, the cells were then incubated with LY379268 (1 µM) for 24 h. The control culture received the same volume of MEM. Cells were collected after incubation and stored at −80°C until use. In another set of experiments secondary astrocytes were exposed for about 1 week to FCS-free MEM containing 0.5% bovine serum albumin (BSA). Afterwards, the cells were incubated with LY379268 (1 µM) for 0–24 h.

Pure neuronal cultures were prepared from striatum of E14-16 C57 Black mice, as previously described [53]. Dissociated striatal cells were grown in 100 mm dishes (Falcon Primaria, Lincoln Park, NJ) using the Neurobasal plating medium supplemented with B27. Cultures were kept at 37°C in a humidified CO_2_ atmosphere and used at 13 DIV. Pure neuronal cultures were incubated with LY379268 (1 µM) for 24 h in the presence or absence of the MEK inhibitor, PD98059 (30 µM), or the PI-3-K inhibitor, LY294002 (30 µM) or the group II antagonist, LY341495 (1 µM). Cells were collected after incubation and stored at −80°C until use.

### In vivo treatments

Adult male C57 Black mice (22–24 g b.w., Charles River, Calco, Verona, Italy) were housed with a 12-h light-dark cycle and food and water ad libitum. All animal experimental procedures were carried out in accordance with the directives of the Italian and European Union regulations for the care and use of experimental animals and were approved by the Italian Ministry of Health.

Groups of 4–5 mice were treated with saline, LY379268 (0.1–3 mg/kg, i.p.) or LY379268+LY341495, the preferential mGlu2/3 receptor antagonist (1 mg/kg, i.p.) to study the expression of GDNF mRNA levels by *in situ* hybridization. A time-course study on GDNF mRNA expression was also performed using LY379268 (0.25 mg/kg, i.p.). Animals were sacrificed at 0, 2, 3 and 6 h under deep anesthesia and brains were rapidly frozen in cooled isopentane and stored at −80°C until used. Other groups of 6–8 animals were injected systemically with either saline, LY379268 (0.25–3 mg/kg, i.p.) or LY379268+LY341495 (1 mg/kg, i.p.), and killed at different times after a single injection. In particular, animals were sacrificed at 3, 6, 12, 24, and 72 h by cervical dislocation. Brains were rapidly removed and cortex and striatum were dissected out and immediately frozen on dry ice and stored at −80°C until used. These animals were utilized for the detection of GDNF mRNA by real-time RT-PCR analysis and GDNF protein levels by Western blot analysis.

Wild-type, *mGlu2^−/−^* and *mGlu3^−/−^* mice were treated with saline or LY379268 (3 mg/kg, i.p.) and killed 24 h later. Both striata were dissected out and immediately homogenized in RIPA buffer for mesurements of GDNF protein levels. The amount of GDNF protein was assessed by Western blot analysis.

Groups of 6 C57 Black mice were treated with three i.p. injections, two h apart, of 24 mg/kg of MPTP (corresponding to 20 mg/kg of free MPTP). Seven days later mice were systemically injected with LY379268, 3 mg/kg, i.p., and killed 24 h later. These mice were used for immunohistochemical analysis of striatal GDNF and glial fibrillary acidic protein (GFAP). Additional group of 8 C57 Black mice were also treated with a single i.p. injection of 36 mg/kg of MPTP (corresponding to 30 mg/kg of free MPTP) and treated with a single i.p. injection of LY379268, 0.25 or 3 mg/kg, 30 min prior to MPTP injecton or injected i.p. daily with LY379268, 0.25 mg/kg. These mice were killed 7 days after MPTP injection and used for assessment of nigro-striatal damage by stereological cell counting of nigral tyrosine hydroxylase (TH)-positive cells. Additional groups of 8 C57 Black mice were treated with a single i.p. injection of 36 mg/kg of MPTP (corresponding to 30 mg/kg of free MPTP) or treated with a single i.p. injection of LY379268, 3 mg/kg, 30 min prior to MPTP injection. Control groups were treated with saline or LY379268, 3 mg/kg, i.p. These mice were killed 1, 2, 3 or 7 days after MPTP injection and used for measurements of GDNF levels in the striatum by ELISA.

Additional groups of 6 C57 Black mice were unilaterally implanted in the left caudate nucleus with pieces of gelfoam (Spongostan, Johnson & Johnson Medical, Milano, Italy, 1.5×1.5×1.5 mm in size) soaked in 5 µl of saline alone (controls) or in 5 µl of saline containing neutralizing anti-GDNF antibody (R&D Systems; 5 µg/gelfoam). The gelfoam was placed in the dorsal striatum underneath the corpus callosum (2.2–3.2 mm depth) under anesthesia with ketamine (100 mg/kg)+xylazine (10 mg/kg), i. p., in a Kopf stereotaxic frame (coordinates: 0.6 mm posterior to the bregma, 1.7 mm lateral to the midline, 2.2 mm ventral from the surface of skull, according to the atlas of Franklin and Paxinos [54]. Twenty-four h later animals were treated with saline or LY379268 (3 mg/kg, i.p.) followed, 30 min later, by a saline injection or a single injection of 36 mg/kg of MPTP (corresponding to 30 mg/kg of free MPTP). Mice were killed 7 days after MPTP injection and used for assessment of nigro-striatal damage by stereological cell counting of nigral TH-positive cells (see below).

### GDNF and NGF probe labelling and in situ hybridization

The GDNF cRNA probe was prepared from a fragment containing 422-bp encompassing nucleotides 279–700 of the originally published GDNF sequence, and cDNA subcloned into the pcDNA3 (Stratagene, San Diego, CA, USA) [55]. The plasmid was linearized with *HindIII* and used as a template for SP6 RNA polymerase to generate the antisense probe or was linearized with *BstxI* and transcribed with T7 RNA polymerase to generate the sense probe. The NGF cRNA probe was prepared from a fragment containing 777-bp of the NGF rat sequence, and cDNA subcloned into the pBSKS (Stratagene, San Diego, CA, USA). The plasmid was linearized with *NcoI* and used as a template for T3 RNA polymerase to generate the antisense probe or was linearized with *EcoRII* and transcribed with T7 RNA polymerase to generate the sense probe.

Serial sagittal (lateral 1.70–1.00 mm) or coronal (A 4.70 mm–5.70 mm) cryostat sections (14 µm) of mouse brain were prepared at striatum level according to the atlas of Lehmann [56]. The *in situ* hybridization procedure was used to examine the expression of GDNF mRNAs in the striatum. Tissue sections were processed for *in situ* hybridization as previously described [57]. Following fixation in 4% paraformaldehyde for 15 min, slides were rinsed twice in phosphate-buffered saline (PBS) and once in distilled water. Tissue was deproteinated in 0.2 M HCl for 10 min, acetylated with 0.25% acetic anhydride in 0.1 M ethanolamine for 20 min, and dehydrated with increasing concentrations of ethanol. Slides were incubated for 16 h in a humidified chamber at 52°C with 8×105 cpm probe in 70 µl hybridization cocktail (50% formamide, 20 mM Tris-HCl pH 7.6, 1 mM EDTA pH 8.0, 0.3 M NaCl, 0.1 M dithiothreitol, 0.5 µg/ml yeast tRNA, 0.1 µg/ml poly-A-RNA, 1x Denhardt's solution, and 10% dextran sulfate), washed twice in 1x SSC (1x SSC = 150 mM NaCl, 15 mM sodium citrate, pH 7.0) at 62°C for 15 min, and then in formamide:SSC (1∶1) at 62°C for 30 min. After an additional wash in 1x SSC at 62°C, single-stranded RNA was digested by RNAse treatment (10 µg/ml) for 30 min at 37°C in 0.5 M NaCl, 20 mM Tris-HCl pH 7.5, 2 mM EDTA. Slides were washed twice with 1x SSC at 62°C for 30 min before dehydration in ethanol and air-drying. For cell localization of mRNA, hybridized sections were coated with NTB Autoradiography Emulsion diluted in water (1∶1) (Eastman-Kodak, Rochester, NY, USA), and stored in desiccated light-tight boxes at 4°C for 4 weeks. Slides were developed with D19 (Eastman-Kodak), fixed with Al-4 (Agfa Gevaert, Kista, Sweden), and counterstained with Cresyl Violet. Semiquantitative data on GDNF mRNA levels in the striatum were obtained by measuring the optical density of the labelling of the autoradiogram films using Image J software (Rasband, WS, ImageJ, U.S. National Institutes of Health, Bethesda, MD, USA, http://rsb.info.nih.gov/ij, 1997–2002), and by evaluation of silver grains over the individual cells from emulsion dipped slides, using image analysis system (IAS-Counter, Delta-Sistemi, Roma, Italy). No labeling was detected with sense ^35^S-labeled riboprobes used as control of the hybridization specificity.

### In situ hybridization and immunohistochemistry

We have used a combination of *in situ* hybridization and immunohistochemical techniques to identify striatal cells expressing GDNF mRNA. Brain cryostat sections were processed for *in situ* hybridization and immunohistochemidtry. Immunohistochemistry labelling was performed immediately after the last washing of the *in situ* hybridization procedure. Sections were washed with PBS, and incubated for 15 min in blocking buffer consisting of 2.5% normal goat serum and 0.3% Triton X-100 in PBS. Subsequently, sections were incubated overnight at 4°C in the presence of the primary antibody diluted in PBS supplemented with 1.5% blocking serum. Mouse monoclonal antibody (1∶400, Sigma, St. Louis, MO), for the detection of glial fibrillary acidic protein (GFAP) and anti-neuron specific DNA-binding protein (NeuN, 1∶400, Chemicon, Temecula, CA, USA) were used as neuronal marker for immunohistochemistry. Sections were then washed three times for 5 min in PBS, and incubated at room temperature for 1 h with a biotinylated antimouse antiserum (Amersham, U.K.), diluted 1∶200. After three 5 min washings with PBS, the sections were incubated for 1 h with a horseradish peroxidase-streptavidin complex (Vector, Burlingame, CA), diluted 1∶100 in PBS. After on washing in PBS and one in Tris-HCl buffer (0.1 M pH 7.4), the peroxidase reaction was developed in the same buffer containing 0.05% 3,3-diaminobenzidine-4 HCl and 0.003% hydrogen peroxide. The reaction was stopped in Tris-HCl buffer and after a short washing with H_2_O, the sections were mounted onto 3-aminopropyl ethoxysilane coated slides dehydrated in an ascending alcohol series, coated in NTB-2 emulsion (Kodak) and processed as described above for autoradiographic development.

### Analysis of GDNF expression by real-time quantitative polymerase chain reaction (PCR)

Total RNA was extracted from mouse striatum and cortex using Trizol reagent (Invitrogen, Milano, Italy) according to manufacturer's instructions. Real-time quantitative PCR was performed using a 2 x Supermix cocktail (Bio-Rad, Hercules, CA, USA) containing the doublestranded DNA-binding fluorescent probe Sybr Green and all necessary components except primers. Quantitative PCR conditions included an initial denaturation step of 94°C for 10 min followed by 40 cycles of 94°C for 15 s, and 55°C for 15 s. Standards, samples and negative controls (no template) were analysed in triplicate. Concentrations of mRNA were calculated from serially diluted standard curves simultaneously amplified with unknown samples and normalized with respect to β-actin mRNA levels. The following primers were used: GDNF, GCCACCATTAAAAGACTGAAAAGG (forward), GCCTGCCGATTCCTCTCTCT (reverse), β-actin, GGTCATCACTATCGGCAAT (forward), GAATGTAGTTTCATGGAATGC (reverse).

### Western blot analysis of GDNF levels

Mouse striatum, cortex and cell cultures were homogenized at 4°C in a buffer composed of Tris-HCl pH 7.4, 10 mM; NaCl, 150 mM; EDTA, 5 mM; PMSF, 10 mM; Triton X-100, 1%; leupeptin, 1 µg/ml; aprotinin, 1 µg/ml. Samples were centrifuged at 12000 *g* for 10 min at 4°C. Equal amounts of proteins (30 µg) from supernatants were separated by 12.5% SDS polyacrilamide gel. After separation, proteins were transferred on immun-blot PVDF membranes. Membranes were incubated overnight at 4°C with a monoclonal anti-human GDNF antibody (1.5 µg/ml, Chemicon International Inc., Temecula, CA) and then incubated for 1 h with the secondary antibody (1∶5000, peroxidase-coupled anti-mouse, Amersham, Milano, Italy). Immunostaining was revealed by the enhanced ECL western blotting analysis system (Amersham, Milano, Italy). The blots were reprobed with anti-β-actin monoclonal antibody (1∶250, Sigma, St. Louis, MO).

### GDNF quantification by ELISA

Mouse striatum was dissected and homogenized in 300 µl lysis buffer at 4°C containing 137 mM NaCl, 20 mM Tris, 1% Nonidet P-40, 10% glycerol, 1 mM phenylmethylsulfonylflouride, 10 µg/ml aprotinin, 1 µg/ml leupeptin, and 0.5 mM sodium orthovanadate and then centrifuged at 10,000 *g* for 10 min at 4°C. The supernatants were removed for ELISA analysis of GDNF, using a commercially available kit (Promega, Madison, WI, USA). Protein content was assessed by the Bradford method. Briefly, 96-well plates were coated with anti-GDNF monoclonal antibody (Promega) overnight. Plates were then washed and nonspecific binding was blocked by incubation with block and sample buffer (Promega) for 1 h at room temperature. Plates were then washed, and 100 µl sample supernatants were added. Plates were then sealed and incubated for 6 h at room temperature. Plates were then washed, polyclonal anti-human GDNF in block and sample buffer was added, and plates were incubated overnight at 4°C. After incubation, plates were washed and then incubated with anti-IGY HRP-conjugated secondary antibody in block and sample buffer for 1 h at room temperature. Plates were then washed, and TMB One solution (Promega) was added to each well for 10 min. The color change reaction was then terminated by addition of 1 N hydrochloric acid, and absorbance was measured at 450 nm using a plate reader.

### Detection of p-ERK1/2 and p-Akt in pure neuronal cultures

Cultured striatal neurons were starved from serum and kept in MS for 24 h; afterwards, they were exposed to LY379268 (1 µM) for 15 min, in the presence or absence of LY341495 (1 µM) at 37°C. Neuronal cells were washed twice with PBS and lysed in Triton X-100 lysis buffer (containing: Tris-HCl, 50 mM, pH 7.5; Triton X-100, 1%; NaCl, 100 mM; EDTA, 5 mM; NaF, 50 mM; β-glycerophosphate, 40 mM; sodium ortovanadate, 200 µM; PMSF, 100 µM; leupeptin, 1 µg/ml; pepstatin A, 1 µg/ml) for 15 min at 4°C. Samples were centrifuged at 12,000 g for 10 min at 4°C. Equal amounts of proteins (100 µg) from supernatants were separated by 12.5% (p-ERK1/2) or 7.5% (p-Akt) SDS-polyacrylamide gel. After separation, proteins were transferred on nitrocellulose membranes. Membranes were incubated with an antibody against phosphorylated extracellular signal regulated kinase, ERK1/2 (phospho-p44/42 MAPK monoclonal antibody, 1∶2000; New England Biolabs, Beverly, MA, USA) for 2 h at room temperature or with an antibody against phosphorylated Akt (1∶1000, rabbit polyclonal phospho-Akt (Ser473) antibody; New England Biolabs) overnight at 4°C. Blots were then incubated for 1 h with the secondary antibody (1∶5000, peroxidase-coupled anti-mouse or 1∶8000, peroxidase-coupled anti-rabbit; Amersham). Immunostaining was revealed by the enhanced ECL western blotting analysis system (Amersham). The same blots were normalized against anti-ERK1/2 or anti-Akt antibodies (1∶1000; New England Biolabs).

### Tyrosine hydroxylase (TH), glial cell line-derived neurotrophic factor (GDNF) and glial fibrillary acidic protein (GFAP) immunostaining

Brains were dissected out and immediately placed in a solution composed of ethyl alcohol (60%), acetic acid (10%) and chloroform (30%). Twenty h later brains were placed in 70% ethanol until they were included in paraffin. Ten µm serial sections were cut and used for histological analysis. Tissue sections were incubated over-night with monoclonal mouse antibody (1∶200; Sigma, St. Louis, MO), or with polyclonal rabbit antibody (1∶20; Santa Cruz Biotechnology, Tebu, France) or monoclonal mouse antibody (1∶400 Sigma, St. Louis, MO), for the detection of TH, GDNF and GFAP, respectively, and then for 1 h with secondary biotin coupled anti-mouse and anti-rabbit (1∶200 Vector laboratories, Burlingame, CA) antibodies for the detection of TH, and fluorescein isothiocyanate-conjugated horse anti-mouse IgG (1∶100; Vector laboratories, Burlingame, CA) for the detection of GDNF and GFAP. Control staining was performed without the primary antibodies.

### Stereological cell counts of TH-positive cells in the substantia nigra pars compacta

The number of TH-positive cells in the pars compacta of the substantia nigra was obtained by stereological technique and the optical fractionator, using a a Zeiss Axio Imager.M1 microscope equipped with a motorized stage and focus control system (Zeta axis) and a digital video camera. The software Image-Pro Plus Windows 6.2 (Media Cybernetics, Inc., Bethesda, MD) was used to control the microscope and to analyze digital images. The analysis was performed on ten sections (10 µm) sampled every 150 µm in a rostro-caudal extension. In each stained section, the area was identified and outlined (magnification of 2.5×). Within each delineated region, neurons were counted (magnification of 100×) according to the optical dissector method counting several boxes (250 µm^2^×2 µm) [58], [59], [60], [61]. The total number of TH-immunoreactive neurons per each rostro-caudal level was computed from the formula: N = Σ(n)×1/SSF×1/ASF×1/TSF) where n is the total number of neurons counted on each dissector, SSF (fraction of sections sampled) is the number of regularly spaced sections used for counts divided by the total number of sections through the substantia nigra pars compacta ( = 1/15); ASF (area sampling frequency) is the dissector area divided by the area between dissectors ( = (2500 µm^2^×dissectors number)/region area) and TSF (thickness sampling frequency) is the dissector thickness divided by the section thickness ( = 2 µm/10 µm). The total number of TH-immunoreactive neurons in the substantia nigra pars compacta is the sum of the total number of TH-immunoreactive neurons per each rostro-caudal level: Ntot = Σ(Ni).

### Quantitative evaluation and statistical analysis

In the *in situ* hybridization study the semiquantitative data of mRNA levels were obtained by measuring the optical density (O.D.) values of the labelling in the film autoradiograms on a personal computer using Image J software. The values for the region measured were defined as those obtained by subtracting the non specific background values. For each experimental group the O.D. values or silver grain counts represent the average of readings from brain sections of four mice. For each experimental condition two independent experiments were performed. The data from *in situ* hybridization or Western blotting were evaluated by one-way ANOVA with intergroup differences analysed by Fisher's Protected Last Significant Difference PLSD test, corrected by Bonferoni's procedure for dependent samples.
