# Correction: Activation of mGlu3 Receptors Stimulates the Production of GDNF in Striatal Neurons

**DOI:** 10.1371/journal.pone.0357247

**Published:** 2026-08-31

**Authors:** Giuseppe Battaglia, Gemma Molinaro, Barbara Riozzi, Marianna Storto, Carla L. Busceti, Paola Spinsanti, Domenico Bucci, Valentina Di Liberto, Giuseppina Mudò, Corrado Corti, Mauro Corsi, Ferdinando Nicoletti, Natale Belluardo, Valeria Bruno

Following the publication of this article [1], concerns were raised regarding Figs 4 and 6.

## Figure 4

The western blot image in Fig 4B includes non-adjacent lanes spliced together, but this is not clearly marked in the panel. The corresponding author stated that lanes in Fig 4B were rearranged relative to the original immunoblot for readability, and that the original immunoblot was loaded in the following sequence: 2 saline-treated wild-type mice, 2 LY379268-treated *mGlu2*^*-/-*^ mice, 3 LY379268-treated wild-type mice, 1 LY379268-treated *mGlu2*^*-/-*^ mouse, 2 saline-treated wild-type mice, and 3 LY379268-treated *mGlu3*^*-/-*^ mice. Lane 2 appears similar to lane 9 in Fig 4B for both β-actin and GDNF bands; the corresponding author indicated that there may have been a mistake in the repositioning of the lanes during figure preparation. The underlying blots for Fig 4B are provided here in S4 File.

The contrast settings appear to be applied differently to the GDNF panel compared to the β-actin panel in the originally published Fig 4B. The corresponding author indicated the contrast may have been modified for clearer data presentation, and that this would not have affected the quantification of the results, which are expressed as the ratio of GDNF/ β-actin. An underlying blot for GDNF expression to support the quantitative data for saline-treated *mGlu2*^*-/-*^ and *mGlu3*^*-/-*^ mice was provided by the corresponding author and reviewed by PLOS. The underlying blot shows that lane 1 in the GDNF panel of Fig 4A originated from a different blot, and that lanes were removed from the GDNF panel of Fig 4A prior to lane 2. The corresponding author requested that this underlying blot not be included with this notice. A graph reporting a new densitometric analysis from the original blots is provided here in S8 File.

## Figure 6

The β-actin panel in Fig 6D is incorrect, and erroneously represents data from the experiment reported in Fig 6C. A revised version of Fig 6 is provided in S1 File where the Fig 6D β-actin panel has been updated with the correct image from the original experiments, and the Fig 6D graph has been updated with a new densitometric analysis from both the original immunoblots and replicate data from the time of the original experiments. Original and replicate data underlying the revised Fig 6 are provided in S1 and S3 Files.

The corresponding author provided additional methodological information for Fig 6D as follows:

The GDNF and β-actin rows in the updated Fig 6D originate from the same single blot. The bands present on the whole membrane were revealed by chemiluminescence captured by film after an exposure time; the same whole membrane was re-probed with anti-actin monoclonal antibody and exposed with a different time to avoid a saturated signal. The whole film was digitally captured to quantitate bands and prepare the final figure. No section of the blot was selectively changed. The new densitometric analysis for the updated Fig 6D (S1 File) was performed using ImageJ software and the data are expressed as GDNF/β-actin ratio. A member of the *PLOS One* Editorial Board reviewed the updated Fig 6D and stated that it appears broadly consistent with the qualitative conclusions presented in [1], and that the updated densitometric values appear to maintain the same overall pattern described in [1]. However, they noted that the β-actin signal in the updated Fig 6D appears relatively strong, potentially approaching saturation, and that the method of between-blot normalization for the new densitometric analysis in the updated Fig 6D pooling data from two experiments is not clear.

The remainder of the underlying data for this article are available from the corresponding author with the exception of some original autoradiographic slices underlying Figs 1C-E, and the original blots for Figs 3A and C, Fig 4A (β-actin and lane 1 for GDNF) and Fig 6A.

## Supporting information

S1 File Underlying blots for Figs 6B-C and the updated Fig 6D, including the replicate experiment from the time of the original experiments used in the densitometric analysis of the updated Fig 6D. (PDF)

S2 File An alternative method for Fig 6D of pooling different immunoblots by expressing data from each single blot as percent of their respective controls, using values of reactive astrocytes not treated with LY379268 (control cultures treated with the same volume of MEM) as controls in the two blots. (PDF)

S3 File Underlying individual-level quantitative data for all charts in Figs 6A-C and the updated Fig 6D. The underlying data for the updated Fig 6D chart contains the new values of GDNF normalized for the correct β-actin panel (Dish #1 and Dish #2) and values from a second, replicate experiment from the time of the original experiments (Dish #3 and Dish #4).
(XLSX)

S4 File Underlying blots for Fig 4B. (PDF)

S5 File Underlying individual-level quantitative data for the chart in Fig 4B. (XLS)

S6 File Underlying images for Figs 1A-B. The Fig 1A panels are derived from GDNF 114 slide and the Fig 1B panels are derived from NGF 115 slide.
(PDF)

S7 File Underlying individual-level quantitative data for the charts in Figs 1C-E. (XLSX)

S8 File Uncropped original film underlying Fig 4B with the original labels for phenotypes and drug treatments, and a graph of a new densitometric analysis of the original blot. (PDF)
