# Supplementary material for: Correction: Activation of mGlu3 Receptors Stimulates the Production of GDNF in Striatal Neurons
Source: PLoS One. 2026 Aug 31;21(8):e0357247. doi: 10.1371/journal.pone.0357247 (PMC13529007; doi:10.1371/journal.pone.0357247)
Supplement: Supplementary file 1 [file pone.0357247.s001.pdf]

FIG. 6B

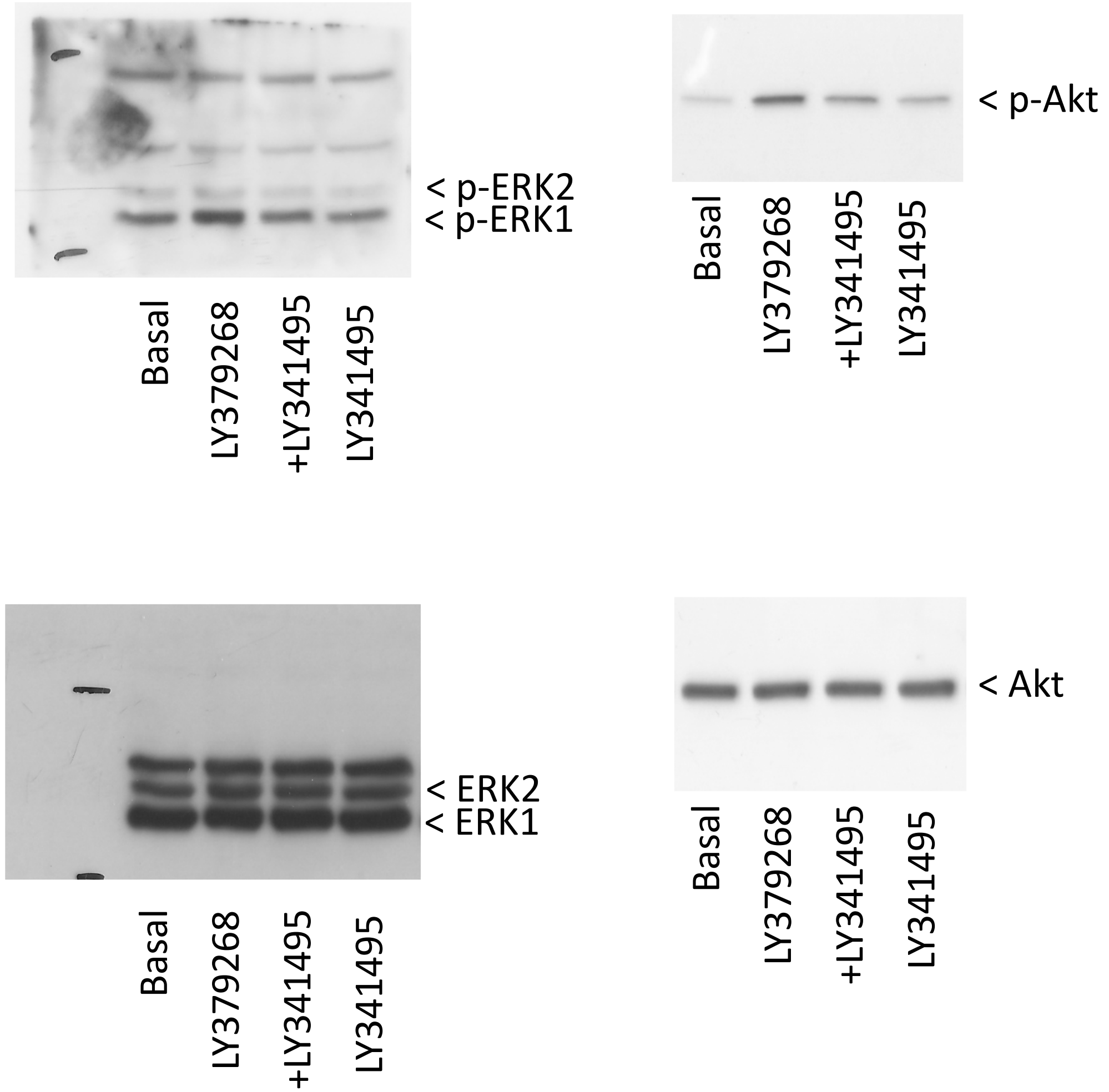

< p-ERK2
< p-ERK1
Basal
LY379268
+LY341495
LY341495
< p-Akt
Basal
LY379268
+LY341495
LY341495
< ERK2
< ERK1
Basal
LY379268
+LY341495
LY341495
< Akt
Basal
LY379268
+LY341495
LY341495

FIG. 6C

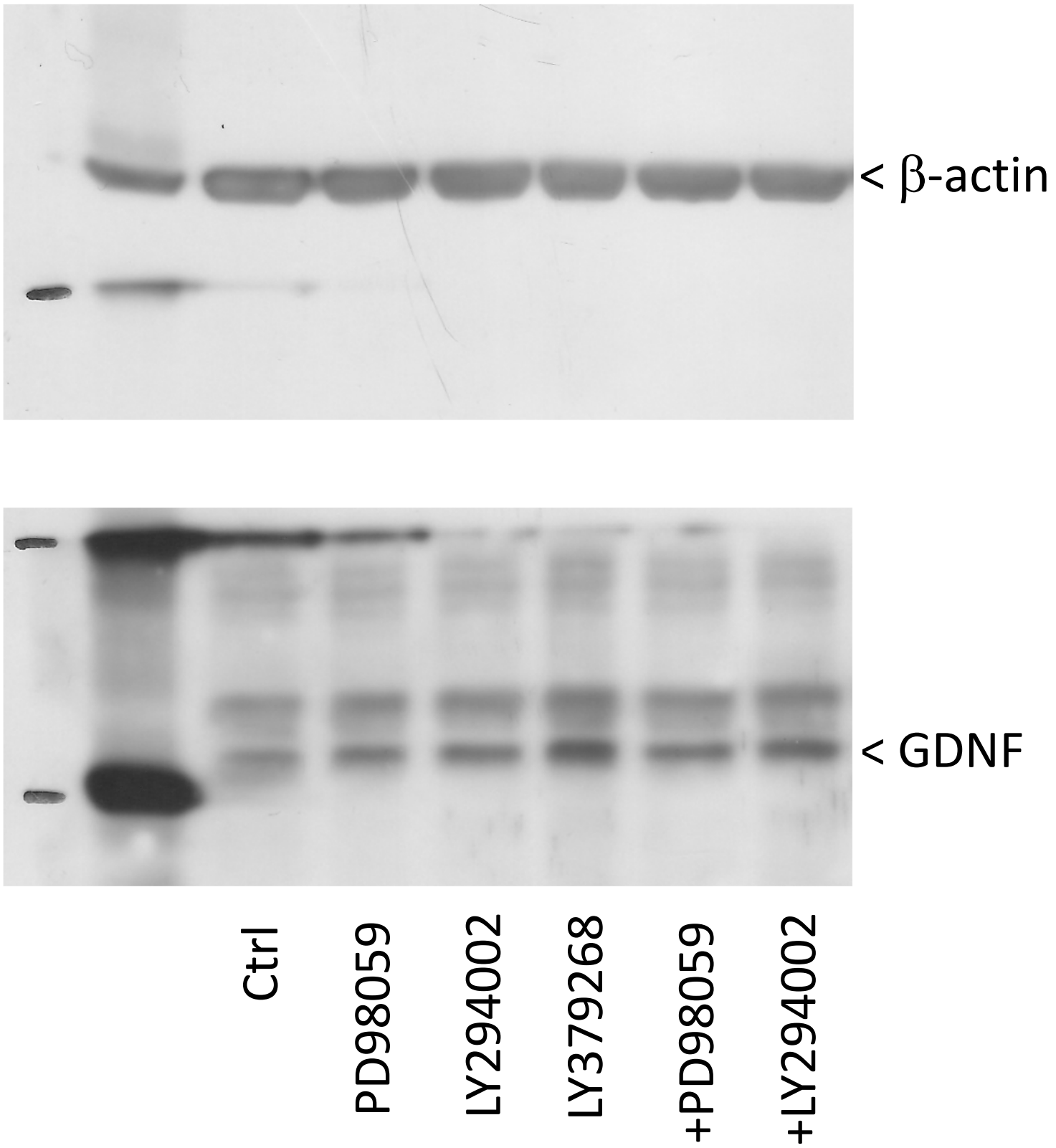

< β-actin
< GDNF
Ctrl
PD98059
LY294002
LY379268
+PD98059
+LY294002

Original blot of Fig. 6D

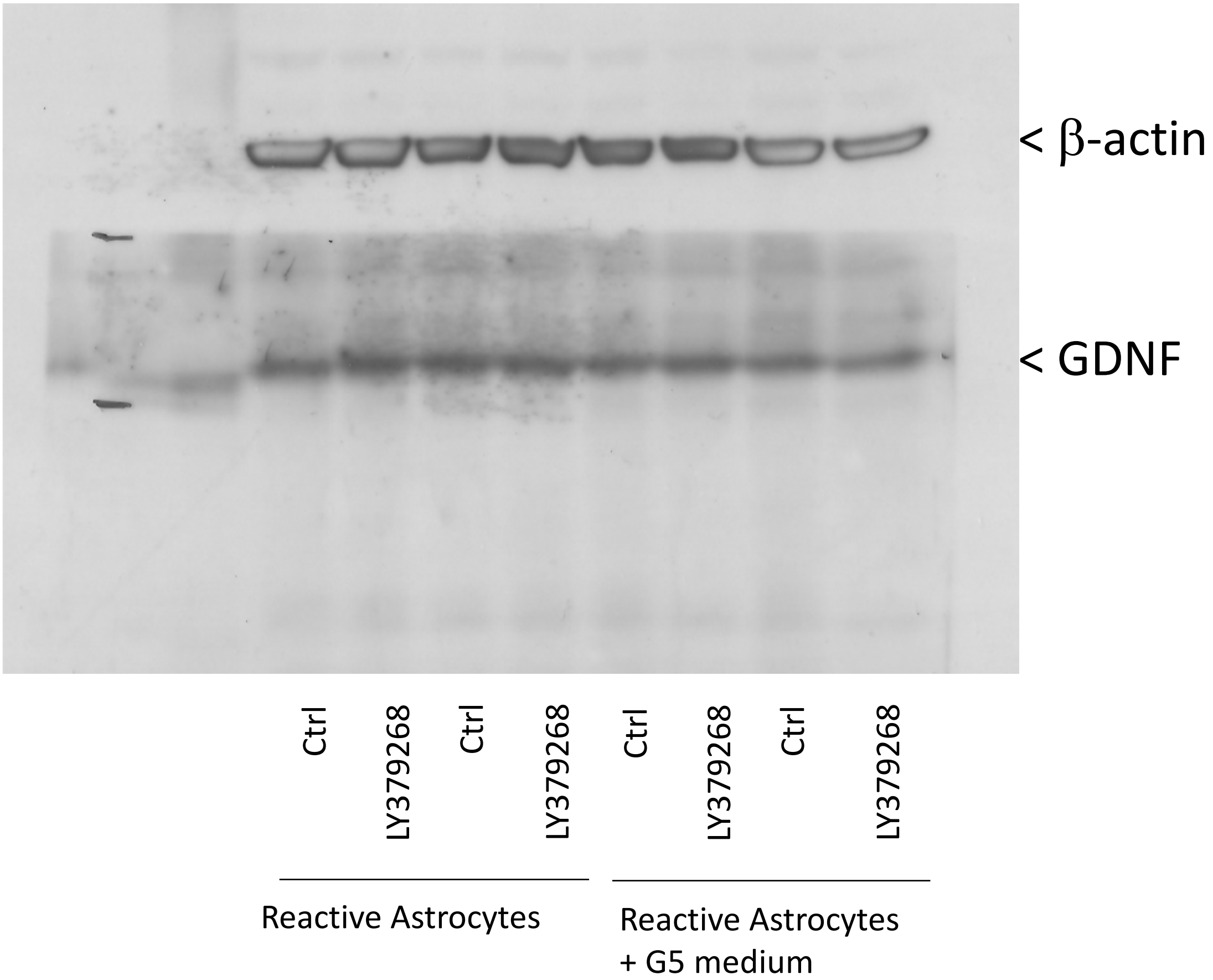

Second blot for densitometric analysis of Fig. 6D

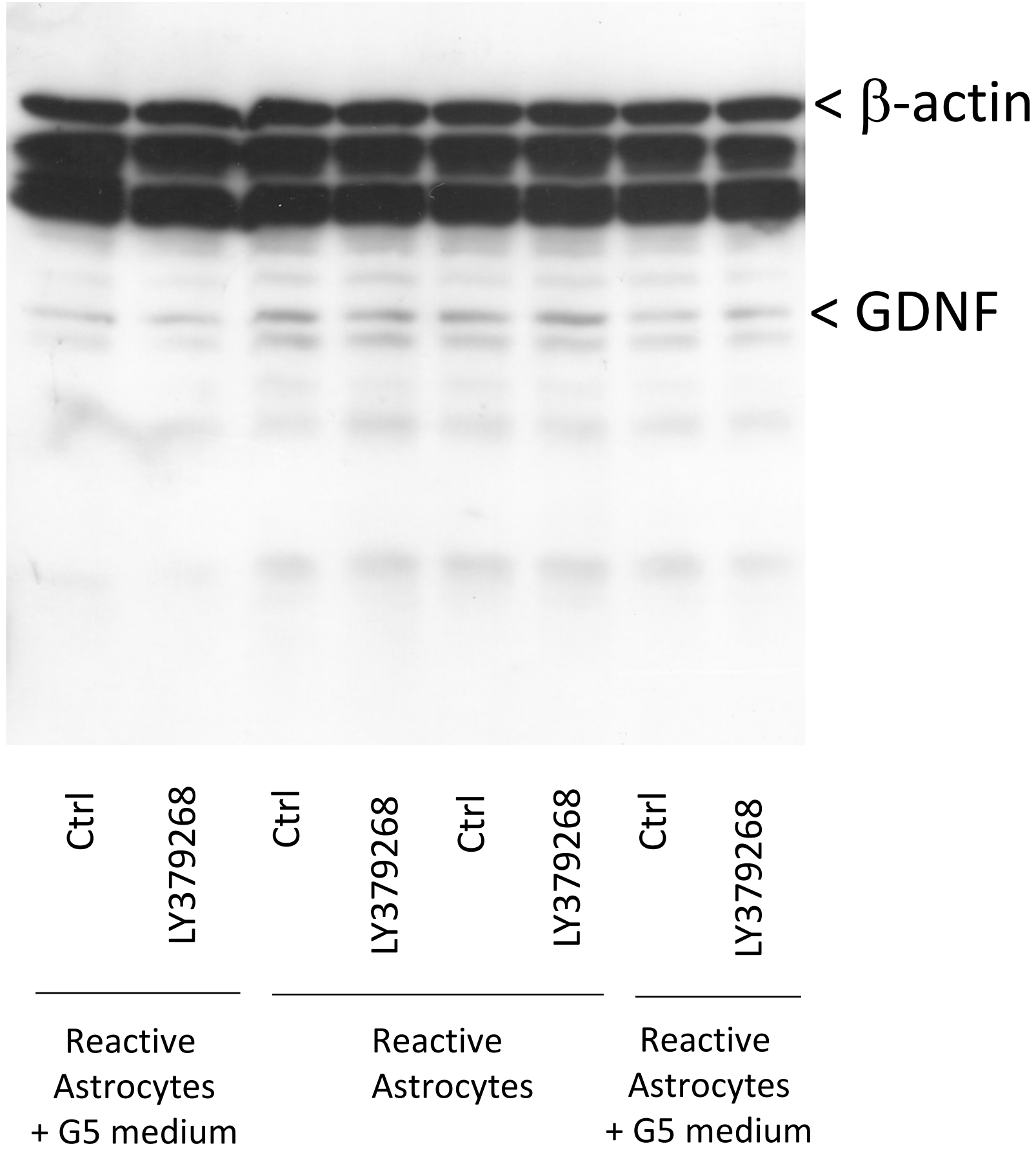

New FIG. 6D

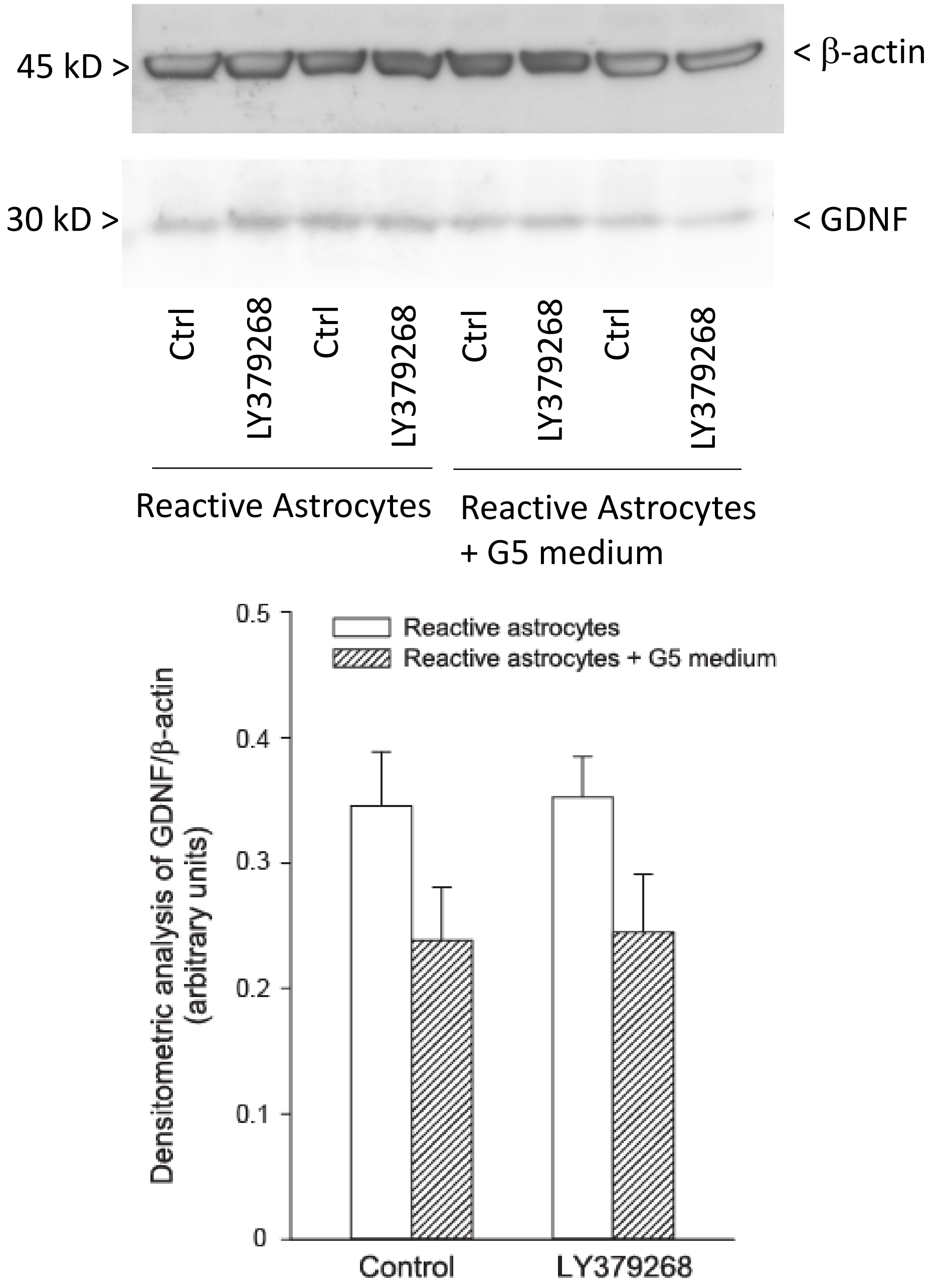

45 kD >
< β-actin
30 kD >
< GDNF
Ctrl
LY379268
Ctrl
LY379268
Ctrl
LY379268
Ctrl
LY379268
Reactive Astrocytes
Reactive Astrocytes + G5 medium
Reactive astrocytes
Reactive astrocytes + G5 medium
Densitometric analysis of GDNF/β-actin (arbitrary units)
0.5
0.4
0.3
0.2
0.1
0
Control
LY379268
