# Supplementary material for: Correction: Activation of mGlu3 Receptors Stimulates the Production of GDNF in Striatal Neurons
Source: PLoS One. 2026 Aug 31;21(8):e0357247. doi: 10.1371/journal.pone.0357247 (PMC13529007; doi:10.1371/journal.pone.0357247)
Supplement: Supplementary file 2 [file pone.0357247.s002.pdf]

New analysis of the data expressed as percent of control reactive astrocytes with no G5 medium (first white column)

Original blot	GDNF	β-actin	GDNF/β-actin	Densitometric analysis of GDNF/β-actin (percent of control reactive astrocytes with no G5 medium)
Reactive Astrocytes (Ctrl)	43	121	**0,36**	**88,88**
Reactive Astrocytes (LY379268)	54	131	**0,41**	**101,23**
Reactive Astrocytes (Ctrl)	55	123	**0,45**	**111,11**
Reactive Astrocytes (LY379268)	50	131	**0,38**	**93,83**
Reactive Astrocytes+G5 medium (Ctrl)	40	134	**0,30**	**74,07**
Reactive Astrocytes+G5 medium (LY379268)	35	111	**0,32**	**79,00**
Reactive Astrocytes+G5 medium (Ctrl)	42	138	**0,30**	**74,07**
Reactive Astrocytes+G5 medium (LY379268)	29	93	**0,31**	**76,54**
**Second blot**				
Reactive Astrocytes (Ctrl)	55	188	**0,29**	**100,00**
Reactive Astrocytes (LY379268)	53	186	**0,28**	**96,55**
Reactive Astrocytes (Ctrl)	52	181	**0,29**	**100,00**
Reactive Astrocytes (LY379268)	59	178	**0,33**	**113,79**
Reactive Astrocytes+G5 medium (Ctrl)	34	187	**0,18**	**62,07**
Reactive Astrocytes+G5 medium (LY379268)	32	190	**0,17**	**58,62**
Reactive Astrocytes+G5 medium (Ctrl)	29	171	**0,17**	**58,62**
Reactive Astrocytes+G5 medium (LY379268)	31	168	**0,18**	**62,07**

In red values of reactive astrocytes not treated with LY379268 (control cultures treated with the same volume of MEM) used as controls.

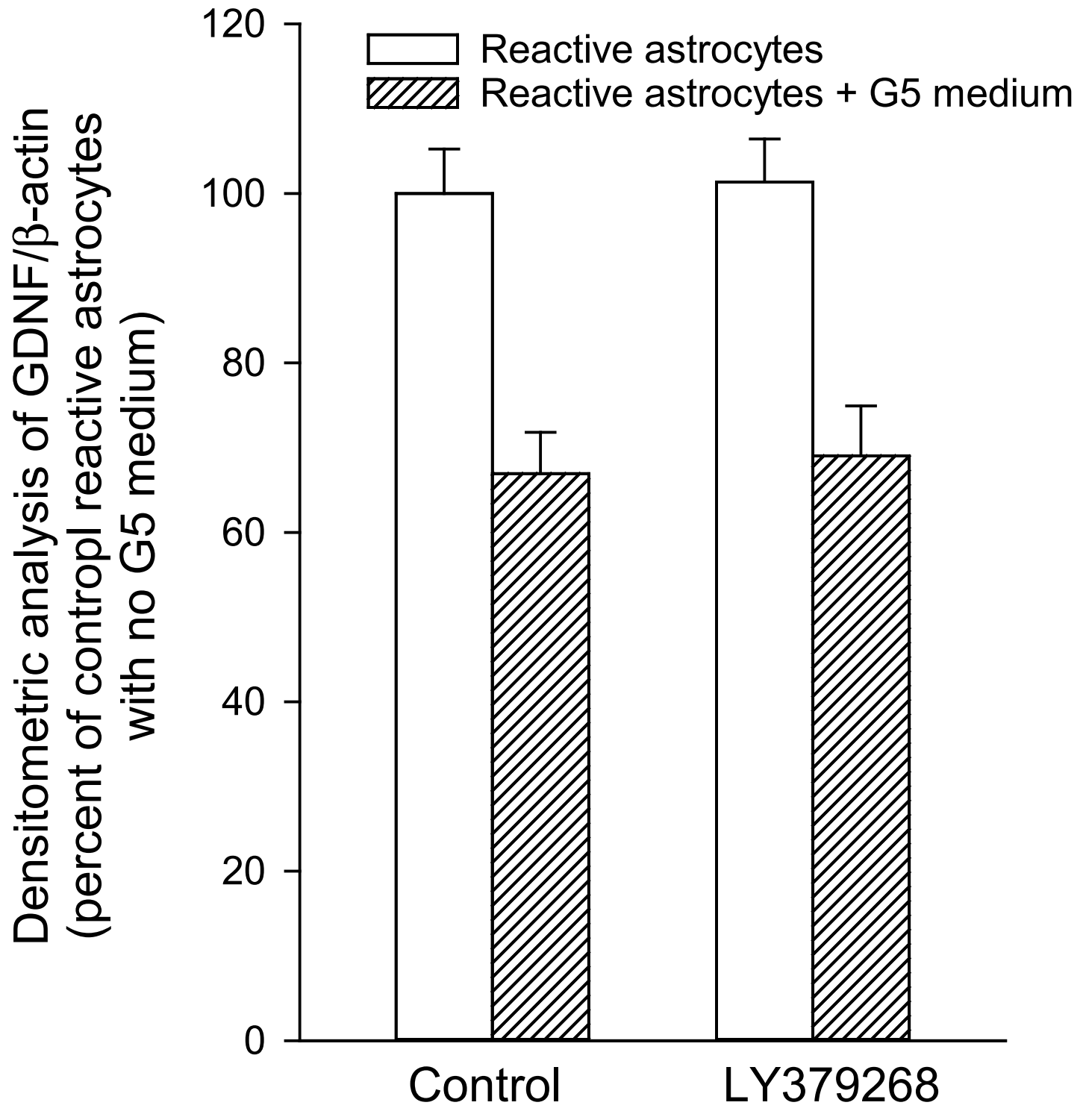

Cumulated densitometric values of the two experiments expressed as percent of control reactive astrocytes with no G5 medium	Mean	S.E.M.
**Reactive Astrocytes (Ctrl)**	**100,00**	**5,24**
**Reactive Astrocytes (LY379268)**	**101,35**	**5,10**
**Reactive Astrocytes+G5 medium (Ctrl)**	**66,96**	**4,86**
**Reactive Astrocytes+G5 medium (LY379268)**	**69,06**	**5,89**
