# Supplementary material for: Correction: Activation of mGlu3 Receptors Stimulates the Production of GDNF in Striatal Neurons
Source: PLoS One. 2026 Aug 31;21(8):e0357247. doi: 10.1371/journal.pone.0357247 (PMC13529007; doi:10.1371/journal.pone.0357247)

Original uncropped
blot of Fig. 4B

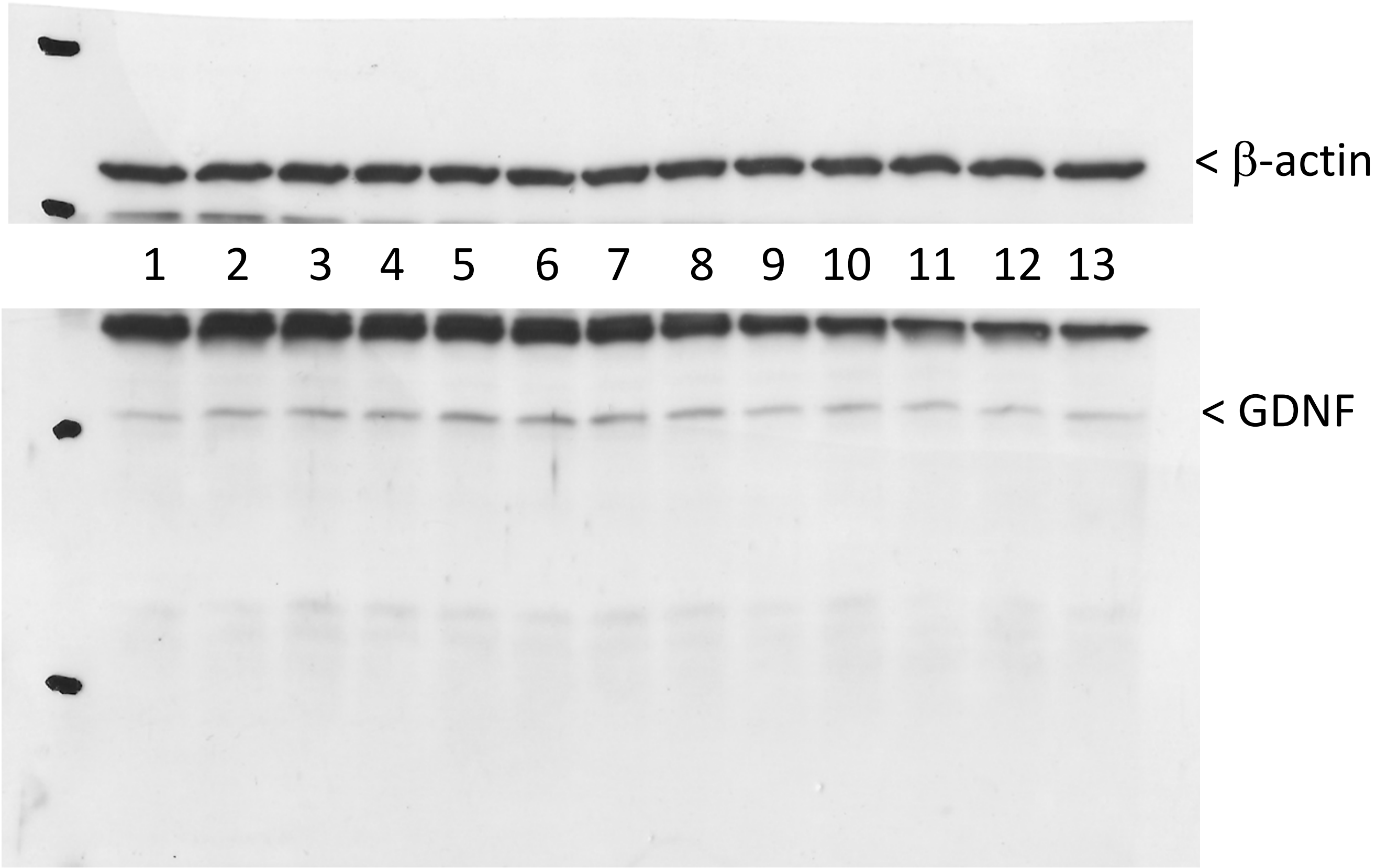


Loading sequence:

1-2 Saline-treated wild-type mice

3-4 LY379268-treated mGlu2$^{-/-}$ mice

5-7 LY379268-treated wild-type mice

8 LY379268-treated mGlu2$^{-/-}$ mouse

9-10 Saline-treated wild-type mice

11-13 LY379268-treated mGlu3$^{-/-}$ mice

Re-arranged blot after easy-to-read
sequencing of the samples

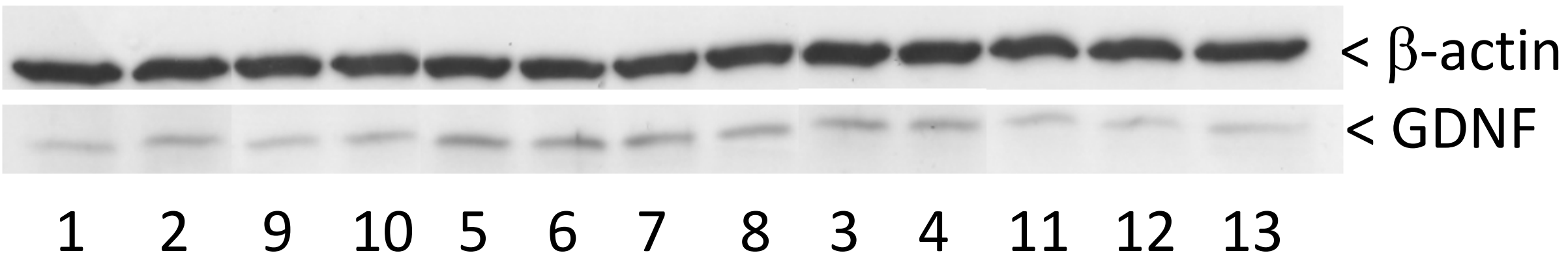

Supplement: Supplementary file 3 [file pone.0357247.s004.pdf]
