# Supplementary material for: Correction: Activation of mGlu3 Receptors Stimulates the Production of GDNF in Striatal Neurons
Source: PLoS One. 2026 Aug 31;21(8):e0357247. doi: 10.1371/journal.pone.0357247 (PMC13529007; doi:10.1371/journal.pone.0357247)
Supplement: Supplementary file 4 [file pone.0357247.s006.pdf]

Original uncropped and unadjusted images

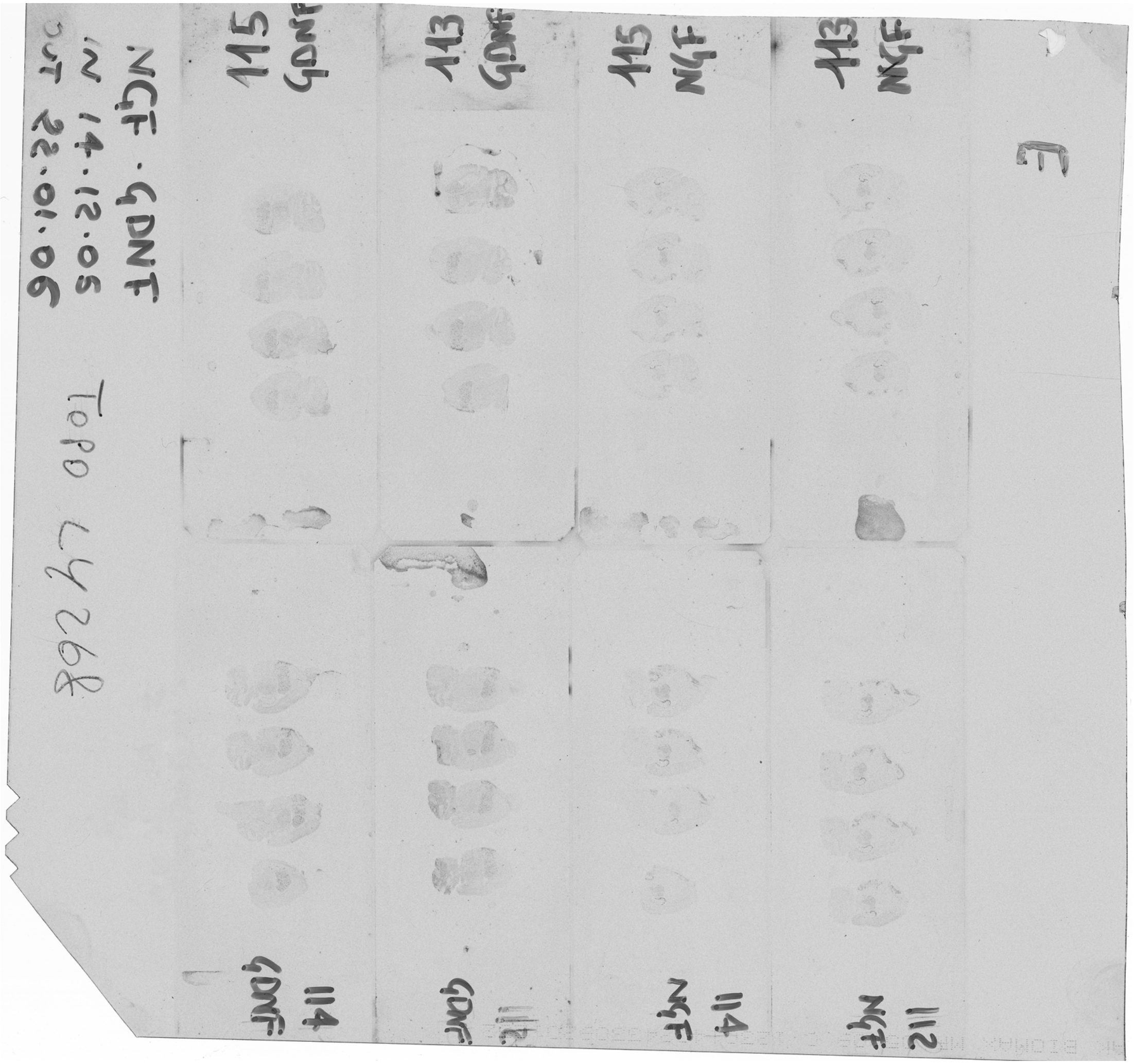

The italian “TOPO LY268” means experiments in which mice were injected with saline or LY379268

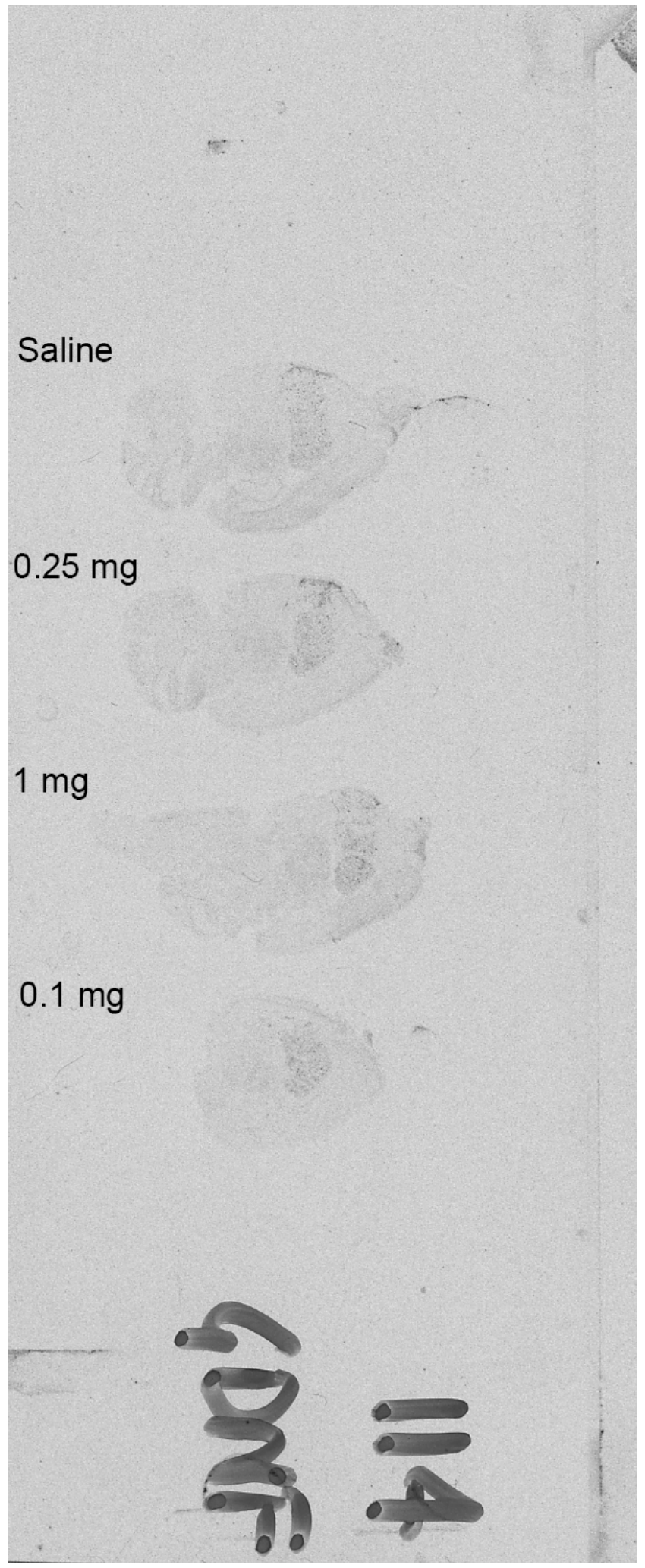

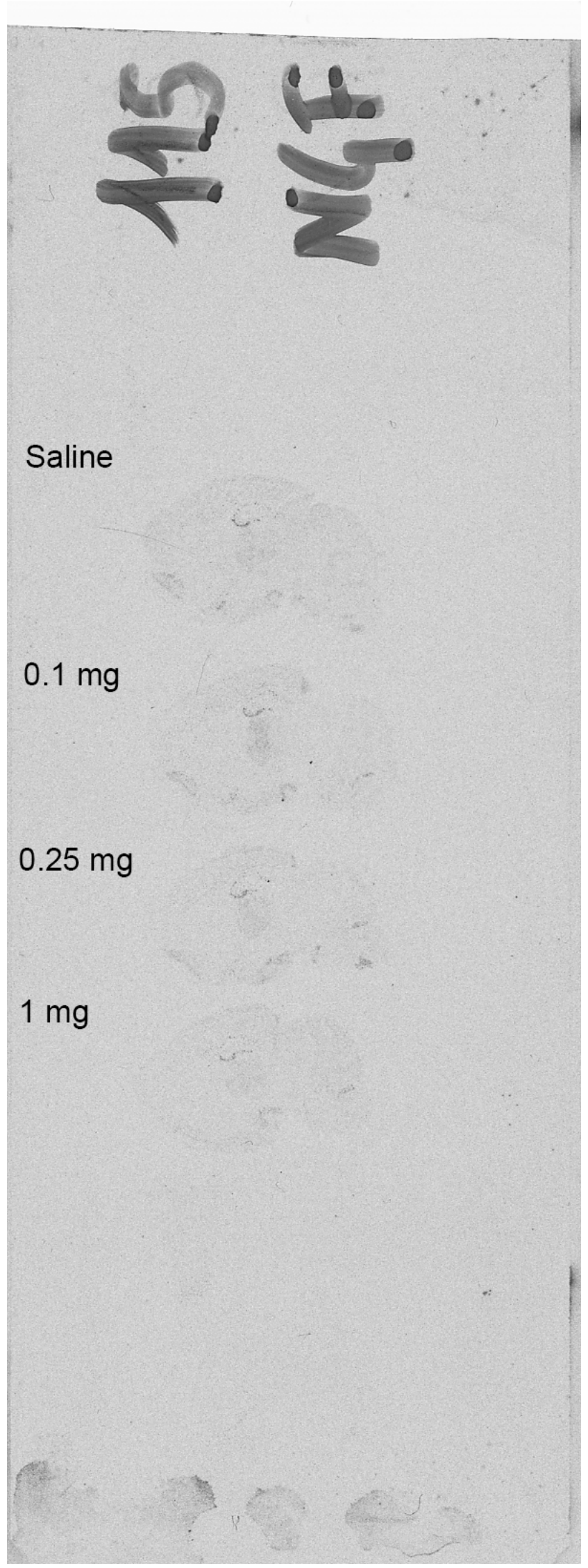

GDNF 114 and NGF 115 were chosen for Fig. 1A and 1B, respectively. Images were uniformely darkened for better visibility.
