# Supplementary material for: Correction: Activation of mGlu3 Receptors Stimulates the Production of GDNF in Striatal Neurons
Source: PLoS One. 2026 Aug 31;21(8):e0357247. doi: 10.1371/journal.pone.0357247 (PMC13529007; doi:10.1371/journal.pone.0357247)
Supplement: Supplementary file 5 [file pone.0357247.s008.pdf]

Original uncropped blot of Fig. 4B showing wild-type mice treated with saline (SAL WT), wild-type mice treated with LY379268 (LY37 WT), mGlu2$^{-/-}$ treated with LY379268 (LY37 KO2) and mGlu3$^{-/-}$ mice treated with LY379268 (LY379268 KO3)

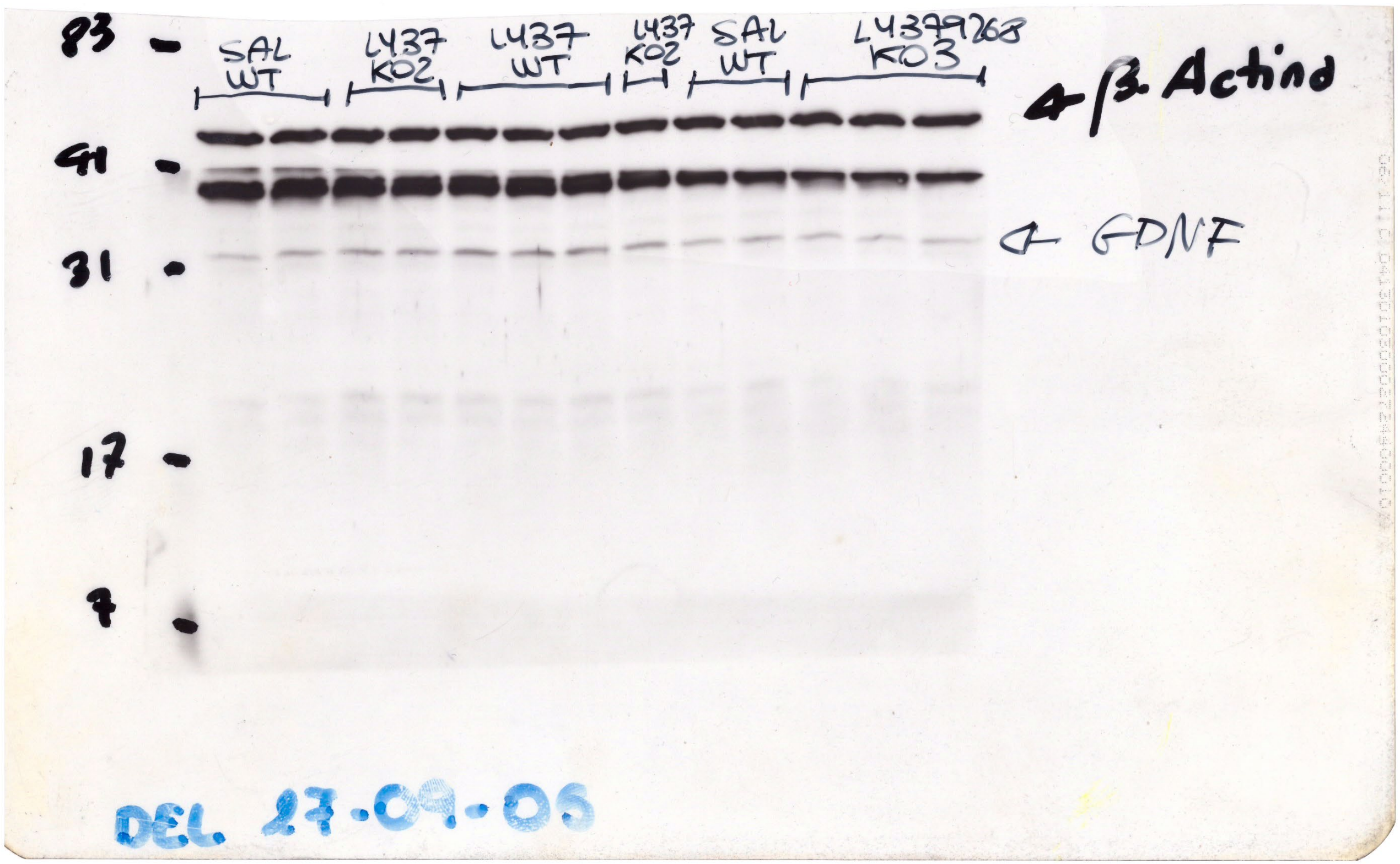


**"DEL 27-09-05" means September 27, 2005**

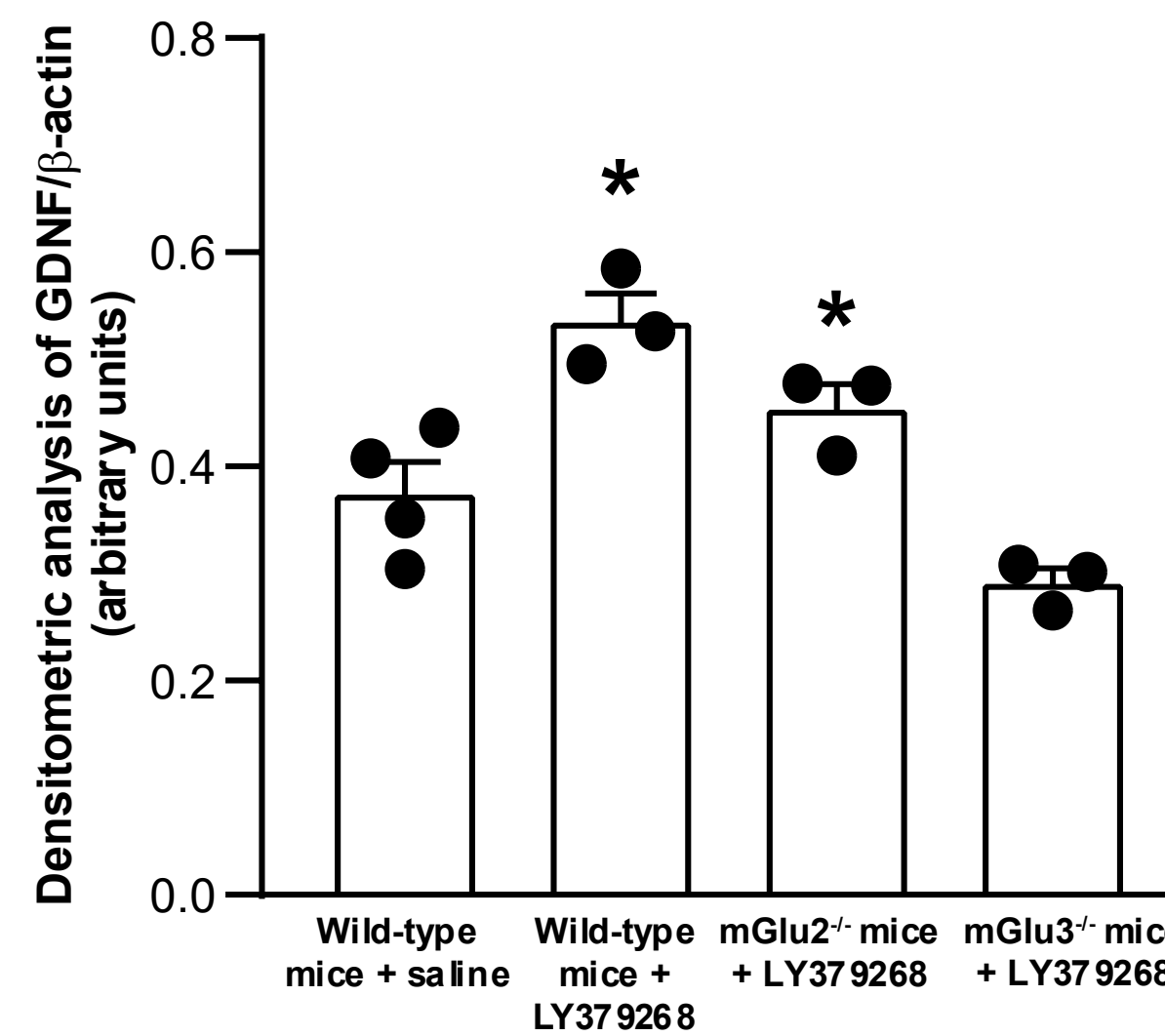


Densitometric analysis of this original blot carried out now.
* $p < 0.05$ (One-way ANOVA + Fisher's PLSD) vs. wild-type mice treated with saline.
